# Cathepsin K: A Versatile Potential Biomarker and Therapeutic Target for Various Cancers

**DOI:** 10.3390/curroncol29080471

**Published:** 2022-08-22

**Authors:** Die Qian, Lisha He, Qing Zhang, Wenqing Li, Dandan Tang, Chunjie Wu, Fei Yang, Ke Li, Hong Zhang

**Affiliations:** 1 Chengdu University of Traditional Chinese Medicine, Chengdu 611137, China; 2 Hospital of Chengdu University of Traditional Chinese Medicine, Chengdu 610075, China; 3 Institute of Interdisciplinary Integrative Medicine Research, Shanghai University of Traditional Chinese Medicine, Shanghai 201203, China

**Keywords:** cathepsin K, CTSK, cancer, biomarker, therapeutic target

## Abstract

Cancer, a common malignant disease, is one of the predominant causes of diseases that lead to death. Additionally, cancer is often detected in advanced stages and cannot be radically cured. Consequently, there is an urgent need for reliable and easily detectable markers to identify and monitor cancer onset and progression as early as possible. Our aim was to systematically review the relevant roles of cathepsin K (CTSK) in various possible cancers in existing studies. CTSK, a well-known key enzyme in the bone resorption process and most studied for its roles in the effective degradation of the bone extracellular matrix, is expressed in various organs. Nowadays, CTSK has been involved in various cancers such as prostate cancer, breast cancer, bone cancer, renal carcinoma, lung cancer and other cancers. In addition, CTSK can promote tumor cells proliferation, invasion and migration, and its mechanism may be related to RANK/RANKL, TGF-β, mTOR and the Wnt/β-catenin signaling pathway. Clinically, some progress has been made with the use of cathepsin K inhibitors in the treatment of certain cancers. This paper reviewed our current understanding of the possible roles of CTSK in various cancers and discussed its potential as a biomarker and/or novel molecular target for various cancers.

## 1. Introduction

It is known that cancer is one of the predominant causes of diseases that lead to death worldwide. In addition, cancers are often definitely diagnosed in advanced stages and cannot be radically cured due to the cancer cells easily metastasizing to other organs of the body [1]. According to the latest epidemic statistics, a total number of 18 million new cancer cases were diagnosed in 2018, and the most common cancer types include lung cancer, breast cancer, liver cancers and other malignant cancers. Cancer is currently the second worldwide cause of death (8.97 million deaths) after ischemic heart disease, but it will likely become the first by 2060 (18.63 million deaths) [2]. Consequently, there is an urgent need for reliable and easily detectable markers to identify and monitor cancer onset and progression as early as possible.

Cancer cells are mutated cells that, in addition to dividing out of control, invade normal surrounding tissues and even migrate to other parts of the body through the circulatory system or lymphatic system. Cancer cells have large nuclei and can proliferate indefinitely, because the adhesion between cancer cells is weakened. It is easy to be agglutinated by lectin, and the adhesion of the wall is reduced, so it promotes its dispersion and metastasis in the body [3]. In cancer therapy, a principal goal is to kill cancer cells while minimizing the death of normal cells [4]. Currently, besides surgery, the main ways for treating malignant cancers include chemotherapy, radiotherapy and biological therapy. However, the therapeutic efficiencies remain unsatisfactory, and therefore, finding new therapeutic targets for cancer treatment has become a prioritized task.

**Figure 1:**
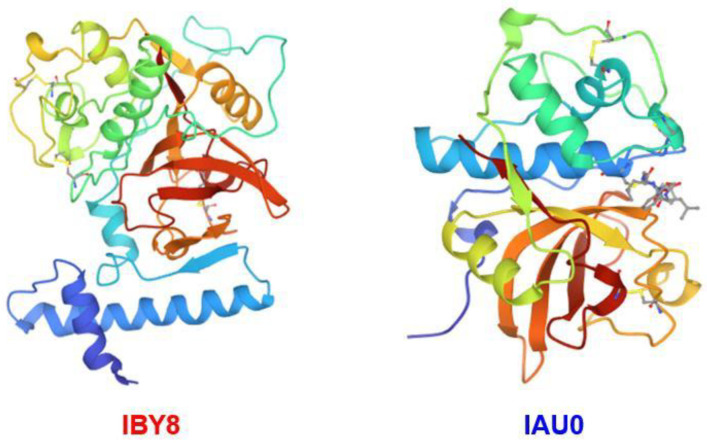
Three-dimensional structure of pro-cathepsin k and cathepsin k. Structures generated on the PDB.

Cysteine cathepsin K (CTSK, Figure 1) is a type of lysosomal cysteine protease that belongs to the papain-like cysteine peptidase family. It is highly expressed in osteoclasts [5,6] and plays a special role in bone resorption [7]. Compared with other cysteines, CTSK is the most active mammalian collagenase [8]. Physiologically, CTSK functions by mediating various aspects of the extracellular matrix turnover, collagen degradation, bone resorption and remodeling of the extracellular matrix, which plays an important role in cancer [9]. CTSK deficiency can lead to severe bone abnormalities, because it is the main peptidase involved in osteoclast bone remodeling [10,11]. CTSK is commonly highly expressed in osteoclasts of bone and would be upregulated under osteoporosis or rheumatoid arthritis; moreover, CTSK overexpression is also found in other tissue lesions, such as malignant cancers and heart failure [12,13,14,15,16]. Increasing reports have suggested that upregulated CTSK could be found in patients with breast cancer [17], giant cell tumors of bone [18], prostate cancer [19] and many other types of epithelial-derived cell cancers [20,21,22]. Increasing studies have reported that the overexpression of CTSK is associated with cancer metastatic disease, indicating its potential diagnostic and prognostic value. In this review, we summarize our current understanding of the role of CTSK in cancer and evaluate its potential as a biomarker and/or novel therapeutic target for cancers.

## 2. CTSK and the Cancers Development

As a cysteine proteolytic enzyme, the hydrolytic activity might play an important role during the development of various cancers. Matrix metalloproteinase (MMP) promotes the cell migration of breast cancer [23] and tumor metastasis in the lungs [24]. CTSK can activate pro-MMP-9 under acidic conditions to produce MMP-9 [25]. In addition, CTSK can degrade the extracellular matrix (ECM) and play a critical role in matrix remodeling [26]. The overexpression of CTSK derived from bone marrow promotes the occurrence of bone metastasis and contributes to the pathogenesis of bone tumor progression [27,28]. The imbalance between the resorption of osteoclasts and the formation of osteoblasts can lead to the occurrence of bone metastatic disease. Especially, tumor cells inhibit the formation of osteoblasts, leading to this imbalance [29]. Tumor cells and multiple myelomas secrete factors that stimulate osteoclast (OC) activity by activating the RANKL/RANK signaling pathway and mediating the resorption of osteoclasts [30,31]. Previous studies have demonstrated that the inhibition of CTSK can significantly decreased the phosphorylation of mTOR at S2448 in Caki cells and reduced tumor growth and induced cell death in a xenograft model [32]. The mTOR signaling pathway plays an important role in maintaining cell growth, proliferation, motility and survival and is involved in the development of a variety of cancers. In addition, the inhibition of mTOR can strengthen the sensitivity of anticancer drugs [33].

## 3. CTSK in Prostate Cancer

**Figure 2:**
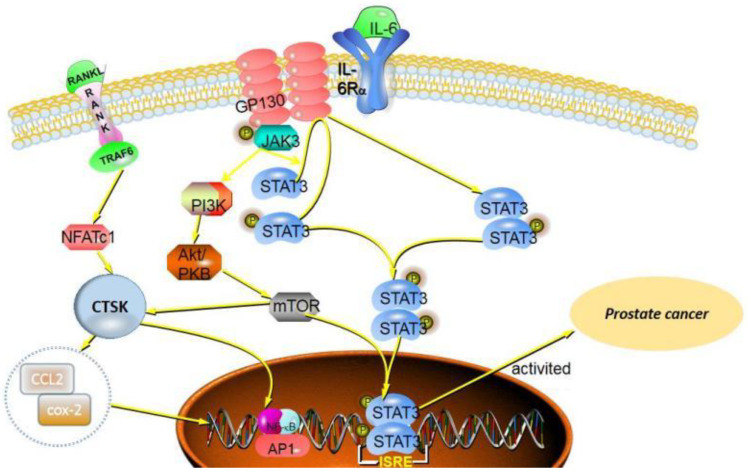
CTSK promotes the invasion and metastasis of prostate cancer, which is related to the receptor activator of NF-κB (RANK) and proinflammatory factors such as interleukin 6 (IL-6).

Recently, large-scale sequencing efforts have allowed a better understanding of the genomic landscape of prostate cancer [34]. Germline or somatic aberrations in DNA damage repair genes are found in 19% of primary prostate cancers and almost 23% of metastatic castration-resistant prostate cancers and compromise the genomic integrity. Patients with BRCA2 pathogenic sequence variants have increased levels of PSA at diagnosis, an increased proportion of high Gleason tumors, elevated rates of nodal and distant metastases and high recurrence rates [35,36]. Bone metastasis is one of the most common complications of PCa in bone microenvironmental components [37]. Currently, studies have revealed that many growth factors such as insulin-like growth factors (IGFs), bone morphogenetic proteins (BMPs), transforming growth factor-beta 1 (TGF-β1) and platelet-derived growth factors (PDGFs) are found in the bone matrix, which participated in the process of bone metastasis [38,39,40]. Moreover, osteoclasts (OCs) are a type of bone cell, and osteoclast resorption is followed by osteocyte apoptosis [41]. It is well-known that the bone matrix components include type I collagen and bone minerals, which can increase bone strength and rigidity [42]. More importantly, CTSK is a key enzyme in the resorption process of osteoclasts and can effectively degrade type I collagen, thereby promoting the bone metastasis of cancers [43] (Figure 2).

## 4. CTSK in Breast Cancer

Breast cancer is the most frequently diagnosed cancer in women and the incidence of female malignant cancers in the first place worldwide [44]. Breast cancer is a heterogeneous disease with multiple histological features and complex molecular mechanisms. The pathology of breast cancer is characterized by the formation of the proliferation of mammary epithelial cells out of control, which is related to a variety of carcinogenic factors. Currently, we have no exact cause for breast cancer, but increasing evidence shows that endogenous estrogens [45], progestogens and other hormones (e.g., androgens and prolactin) [46] are linked to the development of breast cancer. In 2018, Tian et al. demonstrated that estrogen and progesterone promotes breast cancer MCF-7 cell proliferation as a result of inducing the expression of cyclin G1 [47]. In addition, studies have shown that CTSK is strongly expressed in human breast cancers with primary or developing bone metastases [17,48].

Mammary epithelial cells are sheathed by the basement membrane (BM), which is one of the main types of ECM and separates epithelium from stroma and will affect the normal development of glands [49]. Breast cancer is the most prone to bone metastasis cancer. Cintrón et al. [50] studied the effect of ECM on the migration distance of breast cancer MDA-MB-231, and they suggested breast fibroblasts and ECM proteins modulate breast cancer cell migration through MMPs. Approximately two-thirds of patients with advanced breast cancer show osteolytic bone metastases, and RANKL/RANK signaling plays an important role in the migration of breast cancer [51]. RANKL induces the migration of MDA-MB-231, Hs578T and MCF7 breast cancer cells [52]. It is reported that OC-derived bone sialoprotein promotes OC differentiation in an autocrine manner activated by CTSK, resulting in promoting the osteolytic metastasis of breast cancer [53]. Therefore, it is plausible that CTSK has the ability to promote the invasion and migration of breast cancer cells via degrading ECM [17].

**Figure 3:**
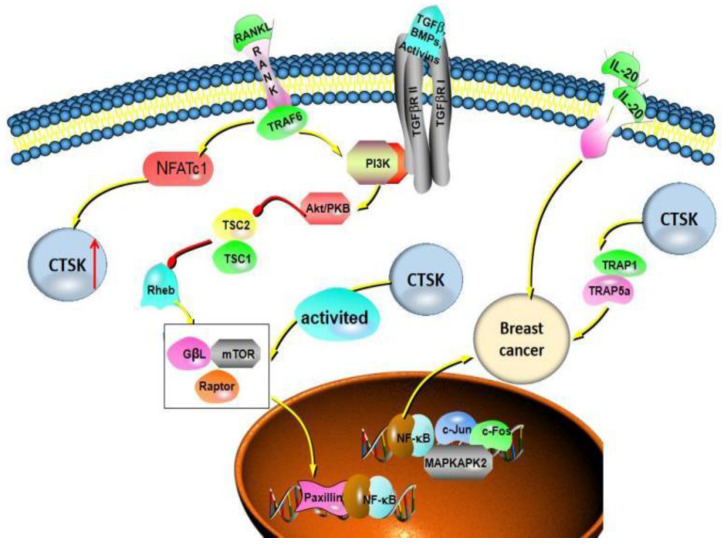
CTSK can promote the proliferation, invasion and migration of different types of breast cancer cells, especially bone metastasis. There are multiple signaling pathways involved in this process, including RANKL/RANK signaling, NF-κB signal pathways, TGF-β and mTOR signal pathways.

In the process of OCs differentiation, both RANKL and TGF-β induce transcription factor NFATc1 to accumulate in the nucleus, resulting in the expressions of the proteins (Src and CTSK) involved in OC-mediated bone resorption [54]. Motyckova et al. suggested CTSK is the transcription target of Mitf and TFE3 through the three consensus elements in the CTSK promoter [55]. The expression of CTSK in breast cancer progression is also associated with other factors. Montgomery et al. reported that, in MDA-MB-231Hi cells, Hyaluronan (HA) induced the overexpression of CTSK and MT1-MMP, and the HA receptor CD44 signal can upregulate cysteine protease CTSK and matrix metalloproteinase MT1-MMP. When CD44 is absent, the expression of CTSK and MT1-MMP is reduced, which reduces the collagen degradation activity of cells and weakens the invasion of cells through collagen [56]. Additionally, Hsu et al. [57] analyzed clinical cases of patients with breast invasive ductal carcinoma, which showed that proinflammatory factor IL-20 is highly expressed in breast cancer bone metastases. IL-20 upregulates CTSK and MMP-9 to promote the proliferation and migration of breast cancer cells in vitro (Figure 3).

## 5. CTSK in Bone Cancer

The giant cell tumor (GCT) of bone is a benign neoplasm of bone with a typical clinical manifestation of eccentric osteolytic lesions. The tumor is characterized by local osteolysis and strong invasiveness [58]. Histologically, the characteristic appearance of GCT shows a large number of osteoclast-like multinucleated giant cells, which resulted in the classification “osteoclastoma” or “giant cell tumor” [59]. Therefore, a giant cell tumor of bone, also known as osteoclastoma, is one of the primary bone tissue cancers. CTSK has a direct effect on bone, degrading the collagen matrix. The research has shown that CTSK is abundantly expressed in multinucleated giant cells and significantly revealed that the CTSK/V-ATPase system is primarily proteolytic factors leading to the osteolysis of GCT [18]. CTSK has a high enzymatic activity, which was more than 100-fold higher than activities found in other tissues expressing CTSK and is mainly responsible for the degradation of the collagen matrix in bone [18]. CTSK plays an important role in maintaining bone homeostasis: the overexpression of CTSK is closely related to the imbalance of bone turnover, leading to bone loss [60]. That is, if CTSK activity is impaired, bone resorption is also impaired. Reportedly, CTSK knockout mice developed profound osteosclerosis [61]. Consequently, based on its important role in bone, CTSK may serve as a potential therapeutic target.

Osteolytic protease mRNA expression profiles revealed that CTSK, cathepsin L and MMP-9 were the preferentially expressed collagenases [18]. The role of CTSK in a giant cell tumor of bone should not be ignored. Recently, the treatment of GCT of bone included surgery and radiation therapy; however, it is more inclined to targeted systemic therapy [62]. RANK–RANKL interaction and macrophage colony-stimulating factor (M-CSF) play important roles in osteoclastogenesis by stimulating the recruitment of osteoclastic cells from blood-born mononuclear osteoclast precursor cells that differentiate into multinucleated osteoclast-like giant cells [63]. The study suggested that denosumab is a fully human monoclonal antibody and is also a RANKL inhibitor that blocks osteoclast maturation and, thus, its osteolytic properties and has become a new treatment option for the locally advanced GCT of bone [64]. In addition, denosumab has been approved by the U.S. Food and Drug Administration for the treatment of adults and skeletally mature adolescents with GCTB that is unresectable or when surgical resection is likely to result in severe morbidity [65]. Interestingly, CTSK is closely associated with RANK–RANKL in the mediation of OC resorption.

## 6. CTSK in Renal Carcinoma

Currently, the incidences of kidney cancer continue to rise worldwide [66]. There are many subtypes of renal cell carcinoma, including clear cell, papillary and chromophobe renal cell carcinoma [67]. The role of CTSK in renal cancers has been extensively studied, since it was first described in a translocated renal cell carcinoma in 2009 [68]. It is reported that almost all Xp11 translocation renal cancers can express CTSK but cannot be expressed in common renal cell carcinoma as clear cell, papillary and chromophobe renal cell carcinomas [69,70]. Increasing researchers have shown that mTOR plays a crucial role in the development of renal cell carcinoma [71,72,73], and CTSK is closely related to the mTOR signaling pathway. Recent studies have revealed that the inhibition of mTORC1 leads to decreased bone resorption and increased bone mass in mice, indicating that it can control osteoclast activities, such as CTSK expressions [13]. Additionally, CTSK inhibitors can significantly reduce the phosphorylation of mTOR in Caki cells [74]. Hence, CTSK plays a key role in the progression of kidney cancers [75,76,77,78].

**Figure 4:**
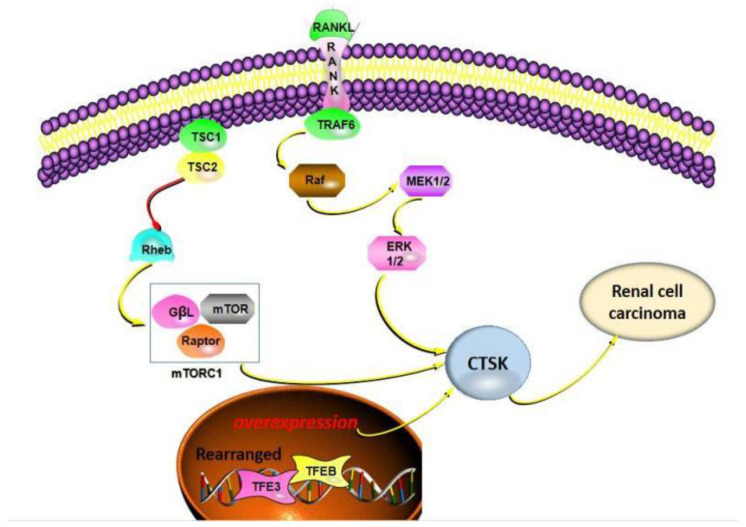
Schematic illustration showing the different mechanisms leading to CTSK expression. Mainly involved in the mTOR signaling pathway, TSC mutations or TFE3 rearrangements or TFEB amplification, which made a vital contribution.

In an ErK rat model of experimental TSC2 gene mutation, CTSK was used as a detection marker for renal cancer cells [79,80]. Moreover, CTSK is positive in eosinophilic solid and cystic renal cell carcinomas, which are relatively rare cancers, and also similar to epithelioid angiomyolipoma [81,82]. Interestingly, TSC mutations or TFE3 rearrangements have been reported in pure epithelioid PEComa/epithelioid angiomyolipoma, and CTSK is also positive [83]. Other studies have also shown that the expression of CTSK is increased in primary renal cell carcinoma [84]. CTSK has been investigated in the primary kidney cancers of primary or transplanted kidneys in patients, and the study regarding urology experts performed a histological examination of renal cell carcinoma, and they also included CTSK as one of the immunodiagnostic signs [85] (Figure 4).

## 7. CTSK in Lung Cancer

**Figure 5:**
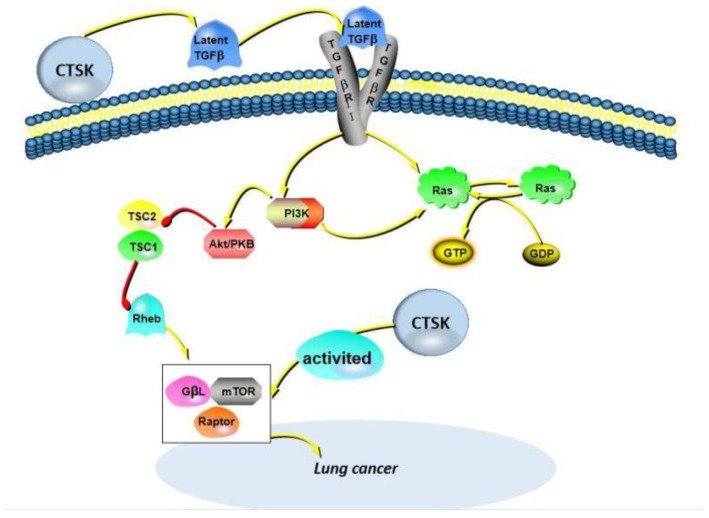
A partial mechanism of action of CTSK in lung cancer. Two pathways of CTSK expression are elucidated: first, transforming growth factor-β (TGF-β) acts as an efficient substrate for CTSK to activate the PI3K and Ras proteins. Second, CTSK is expressed by directly activating the mTOR signaling pathway, mainly the mTORC1 complex.

Lung cancer is the most common primary lung malignant tumor and is commonly divided into small cell lung cancer (SCLC) and non-small cell lung cancer (NSCLC). CTSK is expressed in NSCLC, including adenocarcinoma (ADC), adenosquamous carcinoma, squamous cell carcinoma (SqCC) and large cell carcinoma (LCC), but it is rarely studied in SCLC. As a matter of fact, the expression of CTSK was detected in normal lung tissue, such as bronchial and alveolar epithelial cells, as well as alveolar macrophages [86]. Bühling et al. first reported that CTSK plays a vital effect in pulmonary homeostasis through collagen cleavage via studying on mice and patients with pulmonary fibrosis, which have shown that CTSK has a protective effect, and CTSK expression was significantly upregulated [87]. Zhang et al. [88] found that CTSK is necessary to maintain the structural integrity of the airway by means of the lung airways of wild-type mice. At the same time, TGF-β1 was proven as an efficient substrate of CTSK. Moreover, CTSK was found to be widely and strongly expressed in a pulmonary perivascular epithelioid tumor, which may be a potential biomarker to identify this disease. We focus on reviewing the potential role of CTSK in lung cancer. In 2006, Li et al. [89] reported that osteopontin may promote the migration of osteoclasts by mediating its affinity for CD44 and contains a large number of oblique positive osteoclasts expressing the CD44 immune response. CD44 can promote tumor metastasis, and then, CTSK and MMP-9 were detected, and both were positive. In 2009, Naumnik et al. [90] found that the CTSK inhibitor cystatin C (CystC) concentration was higher than healthy people via the studying of patients with advanced NSCLC. In 2011, Wang et al. reported that the level of CTSK is elevated in tumor-associated macrophages (TAMs) from NSCLC [91]. In 2020, Yang et al. [92] reported CTSK was significantly increased in A549 cells and dispersed in the cytoplasm, and the overexpression of CTSK promotes the proliferation, migration and invasion of A549 cells via the activation of mTOR, which can be blocked by CTSK knockout, suggesting that CTSK may promote the progression of NSCLC by activating the mTOR signaling pathway (Figure 5).

## 8. CTSK in Colorectal Cancer

Colorectal cancer (CRC) is the most common digestive tract cancer in the world [93]. CRC is characterized by diverse molecular features and different cancer stages, with two major mechanisms of genetic instability leading to such changes: chromosomal instability and microsatellite instability (MSI). Although there are challenges in distilling the biological and technical heterogeneity of MSI testing down to usable data, colon cancer screening for defective DNA mismatch repair using the immunohistochemistry and/or MSI test should still be recommended. It has been reported in the literature that immunohistochemistry testing of the mismatch repair machinery may give different results for a given germline mutation and has been suggested that this may be due to somatic mutations [94]. The study showed that DNA somatic copy number alterations, showing common mutations in APC, TP53, KRAS, SMAD4 and PIK3CA, trigger CRC [95]. Furthermore, CRC often arises from the intestinal lining, and its pathological characteristics are complex. Therefore, it is particularly important to explore accurate and convenient diagnostic markers or therapeutic targets.

CTSK has been proposed as a mediator for gut microbiota imbalances causing CRC metastasis, because the serum CTSK levels were significantly higher in a mouse gut microbiota imbalance. A higher CTSK expression in MC38 cells was reported [96], and CTSK was the only upregulated gene in SW480 cells and the RKO cell lines. Recently, Arthur et al. [97] reported that inflammatory factors alter the microbial composition and induce genotoxic microbial expansion to promote tumorigenesis. Additionally, IL-17 increases the levels of cytokines and chemokines produced by myeloid cells; alters the tissue environment and microbiota of CRC and is involved in CRC growth, angiogenesis and metastasis [98]. Interestingly, CTSK could stimulate M2-TAMs to secrete cytokines such as IL10 and IL17 and then promote the invasion and metastasis of CRC cells through the NF-κB pathway [99]. They also demonstrated that tumor-secreted CTSK binds to Toll-like receptor 4 (TLR4) and stimulates the M2 polarization of TAMs through an mTOR-dependent pathway. Clinically, CRC often metastasizes later, which is the main cause of death in patients. As a secretory protein, CTSK was discovered as a novel CRC metastasis pathway, and it is also associated with CRC metastasis and poor prognosis [100]. These results suggest that CTSK is a novel predictive biomarker and a feasible therapeutic target for CRC.

## 9. CTSK in Other Cancers

### 9.1. Ovarian Cancer

Ovarian cancer (OCa) is one of the common malignant cancers of the female reproductive system [101]. Moreover, OCa has often metastasized to other sites. Genomic alterations in the DNA damage repair pathway are emerging as novel targets for the treatment of ovarian cancer. Platinum compounds and PARP inhibitors are the two main classes of drugs active against cancer cells harboring DNA damage repair alterations. On the other hand, as PD-L1 expression remains rare in ovarian cancer, it is necessary to further investigate the potential predictive biomarkers for immune checkpoint inhibitors [102]. According to Xu et al. [103], they reported that the serum levels of CTSK, CA125 and HE4 in OC patients were significantly higher than those in normal controls; compared with the pre-operation, the serum concentrations of CTSK in the samples were reduced by 47% on average after the operation; among the three indicators, the level of decline was higher. In addition, the expression of CTSK in peritoneal metastatic ovarian carcinomas is dramatically higher than that in primary Oca, which is based on immumohistochemical staining results. This makes it possible to diagnose and differentiate between these two types of OCas.

The role of CTSK in OCa has been reported, and Tingting et al. [104] revealed that downregulating CTSK can inhibit cell metastasis and the proliferation of epithelial ovarian cancer cells via suppressing epithelial–mesenchymal transition (EMT). In addition, Zhao et al. [105] revealed that N-myc downstream regulatory gene 1 (NDRG1) can regulate the invasive potential of OCa cells, and their results showed that the downregulation of NDRG1 can also cause the downregulation of CTSK, MMP7and TMPRSS4. In the clinical setting, OCa markers CA125 and HE4 have been widely used [106]. The CA125 glycoprotein commonly appears in epithelial OCa (serous cancers) and has high sensitivity, but its specificity is poor. The study found that CTSK combined with CA125 and HE4 can be more specific to predict OCa with enhanced specificity [103].

### 9.2. Gastric Cancer

Gastric cancer is an idiosyncratic disease, ranking the fourth most common cancer in the world [107]. In addition, patients are usually diagnosed with advanced gastric cancer and have already metastasized, resulting in a low survival rate [26]. Ren et al. [108] reported that CTSK as a downstream factor of cytoskeletal protein coronin 3 promotes gastric cancer metastasis, also including MMP-9. The expression of MMP-9 and CTSK was significantly positively correlated with the expression of coronin 3. It is well-known that tumor cell metastasis is a multi-step process, also called the invasion–metastasis cascade, the first critical step of which is the invasion of the surrounding ECM and stromal cell layers by the primary cancer cells. The study showed that CTSK can rapidly degrade the ECM and promote the metastasis of tumor cells [109]. Moreover, MMP-9 can be cleaved and activated by CTSK in acidic environments, such as tumor and bone resorption [25]. This finding is beneficial to link CTSK to ECM remodeling activated by MMP-9, which may be the key to promoting tumor cell proliferation and migration.

### 9.3. Melanoma

Melanoma, also known as malignant melanoma, is a kind of malignant tumor derived from melanocytes [110]. It is a kind of tumor with a high degree of malignancy and poor prognosis and is prone to distant metastasis [111], and recently, it was published that patients with melanoma of an unknown primary site seem to present better outcomes compared to those with stage-matched melanoma of a known primary site, probably due to the higher immunogenicity, as reflected in the immunologically mediated primary site regression. As such, the melanoma of unknown primary site patients on immunotherapy probably displayed better outcomes when compared to the melanoma of the known primary site subset [112]. What distinguishes melanoma from other cancers is its inherent ability to express melanogenesis enzymes, as well as the corresponding structural proteins to synthesize melanin. 

**Figure 6:**
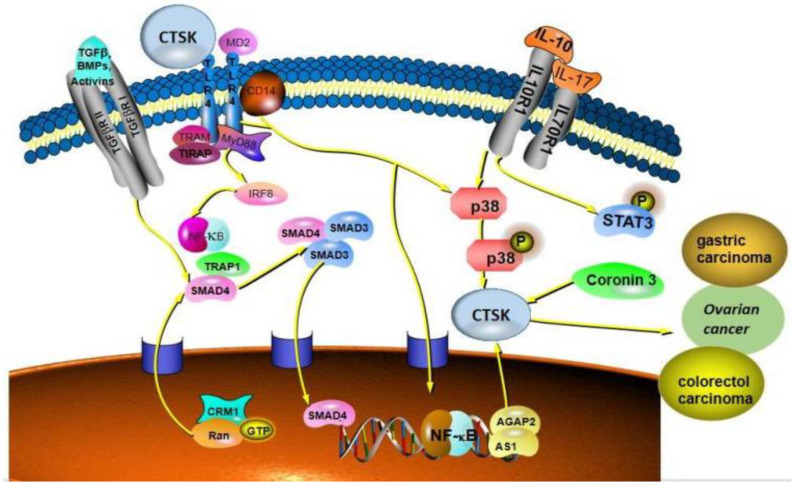
CTSK is strongly expressed in colorectal cancer, ovarian cancer, gastric cancer and melanoma and promotes the invasion and metastasis of these cancers. Mainly associated with the TLR signaling pathway, CTSK binds to the TLR4 receptor and activates the pathway to function.

CTSK was reported to be significantly expressed in the skin and fibroblasts [113]. Studies have shown that CTSK plays a vital role in melanoma cell proliferation, migration and invasion. Quintanilla-Dieck et al. [114] reported that CTSK is positive in most primary melanomas and in all cutaneous melanoma metastases. In turn, the inhibition of CTSK greatly reduced the invasion of melanoma cells through the basement membrane matrix and increased the detection of internalized collagen. Obviously, CTSK enhanced tumor cell invasiveness. Furthermore, Petricevic et al. [115] suggested that CTSK in the primary tumor significantly promotes the occurrence of metastasis and is an independent predictor of the occurrence of metastasis. Melanomas secrete MMP and CTSK through lymph and blood to cut the internal collagen and promote melanoma cells to penetrate the dermis and realize distant metastasis. Then, Rao et al. [116] reported that, using immunohistochemical methods, melanoma patients were studied, and CTSK was measured in comparison with other common markers, such as MITF, HMB45, Melan-A and S100. Their results demonstrated that CTSK is consistently and strongly expressed in melanocytic lesions and has value in distinguishing malignant melanoma from most human cancers. CTSK may mediate the degradation of matrix proteins after phagocytosis in the invasion and metastasis of melanoma. In a word, the role of CTSK in melanoma cannot be ignored and may be a potential target in the future. (Figure 6).

## 10. CTSK Is a Potential Biomarker for Cancers Diagnosis

It is known that most cancer patients are definitely diagnosed at the advanced stage of cancer due to there being inadequate accurate biomarkers in clinical settings to reflect the progress of tumor development. Therefore, specific biomarkers are urgently needed for the diagnosis of tumors in clinical settings [116]. In addition, specific biomarkers can also help to identify high-risk individuals, determine how aggressive a tumor is, whether it has metastasized, closely monitor the tumor progression, develop customized therapies for patients with different cancers and assess the efficacy and outcomes of disease treatment. 

Although, the above-mentioned cancers also have corresponding biomarkers, such as prostate-specific antigen (PSA) for early prostate cancer diagnosis. However, unnecessary biopsies might be needed for the determination, which would lead to numerous complications for patients, such as PSA determination with biopsies resulting in infection, pain, inflammation and hematuresis. Importantly, CTSK has some advantages and specificity in cancer diagnosis, owing to the physiological functions of CTSK, including mediating bone resorption, degrading the extracellular matrix, participating in bone remodeling and other physiological activities. CTSK is commonly highly expressed in osteoclasts of bone and lowly expressed in other organs of normal conditions. However, it would be overexpressed in other tissues due to cancer occurrence.

Currently, due to the lack of specific biomarkers, metastasis of the malignant cancers is often difficult to diagnose. According to previous researchers, it has been reported that the expression level of CTSK is higher after the metastasis of cancer cells. In preclinical or clinical studies, it was found that the high expression of CTSK was detected in the serum and tissues of cancer patients. Bone metastases will occur in most cancers at the advanced stage, causing immeasurable harm to patients. Interestingly, the inhibition of CTSK could be able to reduce the progression of osteolytic lesions, suggesting that CTSK may have greater significance as a tumor biomarker. The CTSK promoter selectively overexpressed breast cancer-associated gene 3 (BCA3), which is a pivotal regulator of NF-κB signaling [117]. CTSK can degrade the extracellular matrix (ECM) to promote the invasion and metastasis of various human cancer types; therefore, the levels of CTSK might be an effective index for assessing the tumor severity or predicting the prognosis of cancers. Bone metastases is a common complication for advanced breast cancer patients with a sharply high mortality. Additionally, bone metastases would induce metastatic bone disease (MBD) with bone pain, fractures and hypercalcemia, which would seriously decrease the life quality of breast cancer patients and increase mortality. In 2010, Jensen et al. reported that the CTSK inhibitor odanacatib (ODN) could inhibit bone resorption in patients with breast cancer and bone metastases, indicating the ODN can be used to treat MBD, and CTSK could be used as a novel therapeutic target for treating MBD [118]. Besides breast cancer, it has been reported that the CTSK inhibitor could suppress the bone metastasis of advanced prostate cancer, and the CTSK inhibitor also diminished prostate cancer-induced bone lesions [37]. A previous study showed that CTSK and TFE3 remain the most sensitive and specific biomarkers for Xp11 translocation renal cell carcinoma [119]. Importantly, it has been reported that several other specific extracellular matrix-associated proteins are highly expressed in cancers, promote the invasion and metastasis of various cancers and have a relationship with CTSK, which may enable CTSK as a new biological marker, providing strong evidence to further improve the tumor identification and diagnostic accuracy. These proteins include MMP-9, which can be used as a biomarker for cancer [120,121,122]; SPARC, an acidic secretory protein rich in cysteine, was positively correlated with CTSK expression and predicted a poor prognosis in prostate cancer and gastric adenocarcinoma [28,95,123]. Tenascin-C (TNC) may also serve as a tumor biomarker that promotes epithelial–mesenchymal transition, proliferation and the migration of cancer cells [124,125,126]. Interestingly, MMP-9 is activated and cleaved by CTSK to function [25], suggesting that CTSK and MMP-9 combinations may be particularly suitable for inclusion in biomarker panels. Additionally, the present study has mentioned that the potential addition of CTSK as a biomarker to diagnose kidneys with clear cell, papillary and eosinophilic features and to assess the severity of the development of renal cancers is exemplified by the findings and distinguished common types of renal cell carcinoma [70]. From all this evidence mentioned above, we have come to the conclusion that CTSK might be a promising specific biomarker for cancers diagnosis combined with other biomarkers or clinical cancer symptoms. We summarized the effects and mechanisms of CTSK in some cancers, as shown in Table 1.

## 11. CTSK Is a Potential Therapeutic Target for Cancers

This study found that tumor-derived exosomes not only promote tumor invasion and metastasis but also inhibit osteoclast differentiation, and CTSK expression is downregulated [136,137]. This suggests that there is some connection between the role of exosomes in CTSK and cancer. However, in this manuscript, we focus on the role of CTSK in cancer. It is known that some biomarkers are also the therapeutic target for cancers, and searching novel CTSK inhibitors might be beneficial for the treatment and prevention of various cancers. As mentioned above, CTSK is involved in the pathological process of cancer development, so it seems that CTSK could be also a potential therapeutic target for cancer treatment. There is no doubt that CTSK is a specific therapeutic target of osteoclasts [138,139]. However, increasing current studies have also suggested that some non-osteoclast cell types also express CTSK, which would be upregulated when the cancers occurred. CTSK has the powerful ability to degrade the extracellular matrix (ECM), which would be beneficial for the metastasis of various human cancer types, including breast, lung, prostate and melanoma [140]. Additionally, it was also reported that CTSK can promote the proliferation and metastasis of cancer cells via activating the PAR-3 and PAR-4 receptors.

In the acidified environment of the bone resorption cavity, CTSK hydrolase could also degrade the type I collagen and bone organic matrix, which can increase the bone metastasis of various human cancers. During bone resorption, CTSK-positive cells accumulate at the tumor–bone interface until the bone is destroyed. It is the mechanism by which CTSK mediates osteoclast resorption. Additionally, in areas of active bone turnover, both bone formation and bone resorption promote tumor cell proliferation [141]. In addition, it was reported that 70% of patients with metastatic prostate and breast cancers have bone metastases, and bone is a common site of metastasis in most solid cancers [142]. Grabowskal et al. [143] reported that CTSK inhibitors are the first drugs to prevent bone resorption without affecting bone formation, thereby improving bone quality and strength. Given the critical role of CTSK in bone remodeling, CTSK inhibitors should benefit the treatment of cancers characterized by extracellular matrix degradation. For instance, in animal experiments studying breast cancer, the intravenous inoculation of CTSK-expressing human B02 breast cancer cells resulted in the formation of bone metastases. Le et al. [48] revealed that CTSK may be a therapeutic target for bone metastases, because the use of CTSK inhibitor CKI can reduce the progression of osteolytic lesions and inhibit osteoclast resorption, thereby reducing the skeletal muscle tumor burden. Vashum et al. [144] indicated that ODN has no effect on the osteoclast number, and it was proven that ODN prevents osteolytic metastasis by interacting with the NF-κB pathway to inhibit bone resorption cytokines and growth factors. The CatK inhibitor L-235 (100 mg/kg) significantly reduced the intratibial tumor volume by 63% and is more effective than 56% zoledronic acid (ZOL) in reducing breast cancer invasion [145]. Liang et al. [146] studied the effects of CTSK inhibitors on prostate cancer in mice. There are three main effects of CTSK inhibitors: inhibition of PCa cell invasiveness, dose-dependent inhibition of PCa-conditioned mediator-induced bone resorption and the effective prevention of tumor formation. Thus, selective CTSK inhibitors may prevent the establishment and progression of breast cancer and prostate cancer in the bone, becoming a new treatment for advanced cancers. In addition, CTSK has also shown its potential as a therapeutic target in lung cancer and melanoma and has been clinically validated [92,115,147]. Clinically, this study on healthy volunteers showed that the adverse reactions of a single dose of ODN are transient, mild to moderate and commonly headache and gastroenteritis, with a half-life period of 40–80 h, indicating that it is well-tolerated and has efficacy [148]. Consequently, CTSK may serve as a versatile therapeutic target for various human cancers, and the inhibition of CTSK is of great significance for the treatment of cancers.

Currently, there are a variety of CTSK inhibitors reported in the previous literature (Table 2), and some of them have been studied in preclinical studies, such as relacatib (SB-462795) [149], L-235 [150], balicatib (AAE-581) [151], odanacatib [152] and ONO-5334 [153]. They are taken orally and are able to significantly reduce the biochemical markers of bone resorption, serum CTX and urinary NTX levels and increase the bone mineral density. Of note, among the CTSK inhibitors, only odanacatib has been studied in the setting of a phase III clinical trial [154]. Therefore, we will focus on odanacatib-related research. ODN is a selective and reversible inhibitor of CTSK. By studying ovariectomized adult rhesus monkeys, it was shown that ODN increased the bone mass, improved bone strength at the hip and displayed site-specific effects on this process [152]. The inhibition of CTSK achieves the balance of bone turnover and reduces bone loss [155]. Additionally, in postmenopausal women administered 50 mg ODN, reductions in the serum CTx of −66%, and urine NTx/creatinine (uNTx/Cr) of −51% relative to the placebo were observed at 24 h. At 168 h, reductions in the serum CTx (−70%) and uNTx/Cr (−78%) were observed relative to the baseline [148]. It has a long metabolic half-life in vivo and a low clearance rate, indicating that it can exert a long-term drug effect [156]. The cross-linked N-terminal (NTx) and C-terminal (CTx) telopeptides of type I collagen are generated by the enzymatic action of CTSK on collagen. CTSK cleaves the N-telopeptide of collagen to generate NTx. CTx and NTx were used throughout phases I, II and III of ODN clinical studies [157]. Therefore, CTSK effects can be assessed by detecting CTx in the serum and NTx in urine [158,159]. To sum up the above, CTSK plays an important regulatory role in tumor invasion and metastasis by degrading the collagen matrix.

Here, we need to point out a fact. Owing to its prominent role in bone resorption, CTSK has become a therapeutic target for osteoporosis [160]. In fact, CTSK not only plays an important role in bone, but its activity has profound effects on various organs beyond bone. Evidence from human and animal studies has documented that CTSK is involved in the development of the central nervous system, cardiovascular system, respiratory system and related diseases [161]. For example, according to the study by Hua et al., the knockout of CTSK can reduce cardiac hypertrophy caused by a pressure overload, suggesting that CTSK may serve as its therapeutic target [162]. Furthermore, combined bone metastases are a common and devastating consequence of most cancers. Based on recent research findings, this article reviews the role of CTSK in various cancers, which is expected to be a potential therapeutic target. Cancer growth in bone requires the activation of various signaling pathways in cancer cells and stromal cells, including those stimulated by TGF-β and RANKL and mediated through Src tyrosine kinase, mTOR signaling mediated by mutations in TSC2 and the Wnt/β-catenin pathway [79,163]. CTSK is associated with the activation of these signaling pathways and is arguably a novel target for cancer-induced bone resorption. The specific and clear mechanisms of the effects related to CTSK needs to be verified by further experiments.

**Table 2:** The reported potential inhibitors of CTSK.

Compound	Formula	Chemical Structure	Bioactivity	Reference
Odanacatib	C25H27F4N3O3S	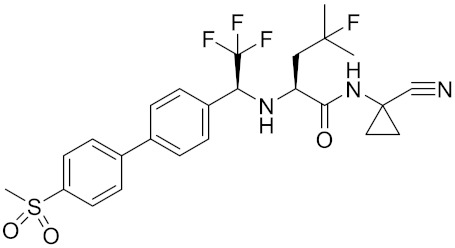	Inhibitor, IC50 = 0.2 nM (Human), IC50 = 1 nM (Rabbit); odanacatib (30 mg/kg/day, orally) can persistently suppress bone resorption in OVX monkeys.	[155,164,165]
MK-0674	C26H27F6N3O2	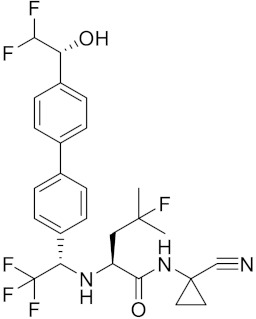	Inhibitor, IC50 = 0.4 nM	[166]
L-873724	C23H26F3N3O3S	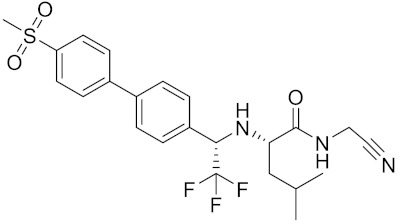	Inhibitor, IC50 = 0.2 nM	[167,168]
Balicatib/AAE581	C23H33N5O2	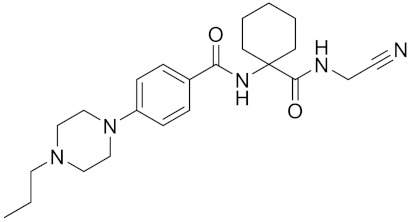	Inhibitor, IC50 = 1.4 nM; IC50 = 56 nM (Rat); IC50 = 480 nM (mouse)	[169]
NC-2300/VEL-0230	C14H24NO5 -	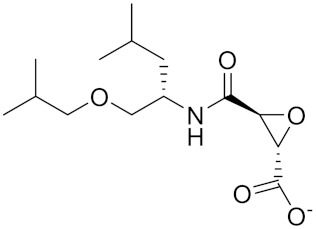	Inhibitor, IC50 = 46 nM; IC50 = 319 nM (Rat); IC50 = 102 nM (mouse)	[169]
Gü1303	C20H22N4O3	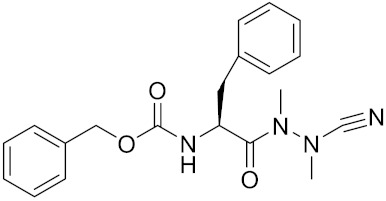	Inhibitor, Ki = 0.91 nM	[170]
Gü2602	C16H22N4O3	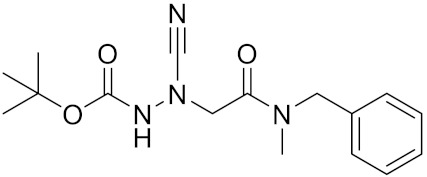	Inhibitor, Ki = 0.013 nM	[170]
Cathepsin K inhibitor 2	C30H33F4N5O3	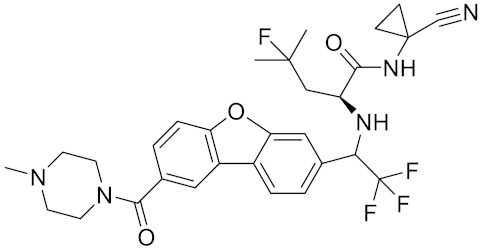		[171]
Cathepsin inhibitor 1	C20H24ClN5O2	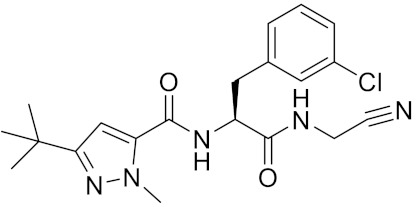	Inhibitor, IC50 = 5.5 nM	[172]
Relacatib/ SB-462795	C27H32N4O6S	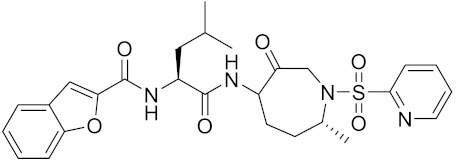	Inhibitor, Ki = 0.041 nM	[149,173]
BML-244	C11H21NO3	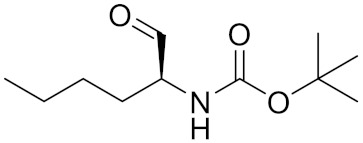	Inhibitor, 1 μM	[174]
4S-7-cis-methylazepanone	C27H32N4O6S	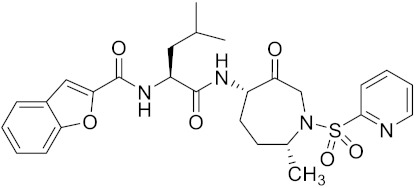	Inhibitor, Ki = 0.16 nM (human)	[173]
4S-parent azepanone	C26H30N4O6S	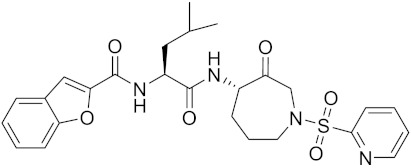	Inhibitor, Ki = 0.16 nM (human)	[173,175]
Compound 24	C40H47N5O7	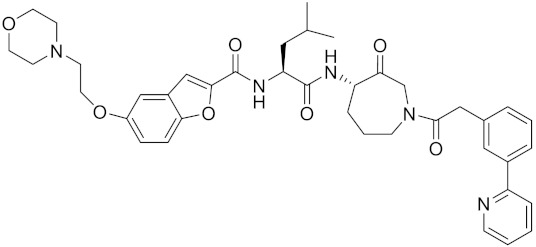	Inhibitor, Ki = 0.0048 nM (human); Ki = 4.8 nM (rat)	[175]
ONO-5334	C21H34N4O4S	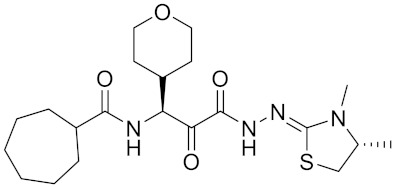	Inhibitor, Ki = 0.10 nM (human); Ki = 0.049 nM (rabbit); Ki = 0.85 nM (rat)	[176,177]
2-Cyanopyrimidine	C5H3N3	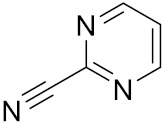	Inhibitor, IC50 = 170 nM	[178]
LHVS	C28H37N3O5S	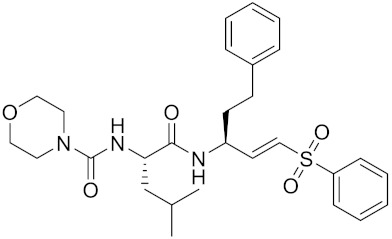	Inhibitor, 5 μM (Osteoclasts)	[179]
L-006235/L-235	C24H30N6O2S	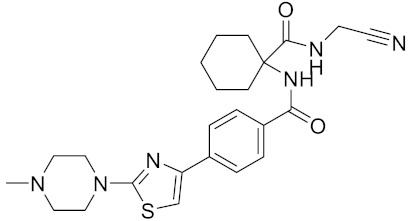	Inhibitor, IC50 = 5 nM	[142,150,180]
calpeptin 1/Cbz-Leu-Nle-H	C20H30N2O4	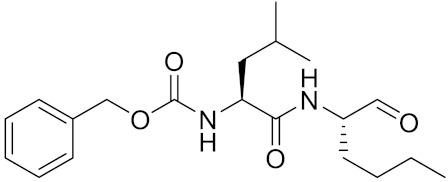	Inhibitor, IC50 = 0.11 nM	[181]
Boc-Nle-H	C11H21NO3	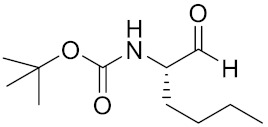	Inhibitor, IC50 = 51 nM	[181]
Inhibitor 9		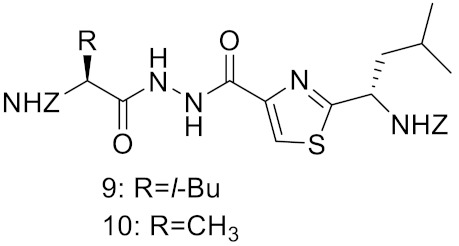	Inhibitor, Ki = 10 nM	[182]
Inhibitor10			Inhibitor, Ki = 120 nM	[182]
Compound rac-34a	C22H23F2N2OS	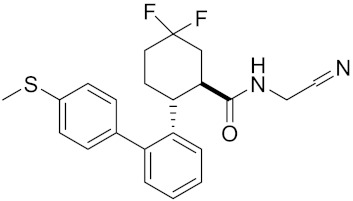	Inhibitor, IC50 = 0.46 nM	[183]
Compound (−)34a			Inhibitor, IC50 = 0.28	
Compound (+)34a			Inhibitor, IC50 = 7.1	
Compound rac-34b	C21H19F3N2O	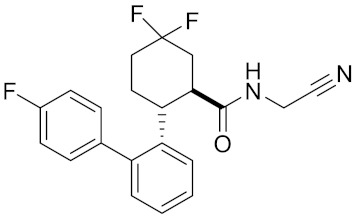	Inhibitor, IC50 = 36	[183]
Compound rac-34c	C22H22Cl2N2OS	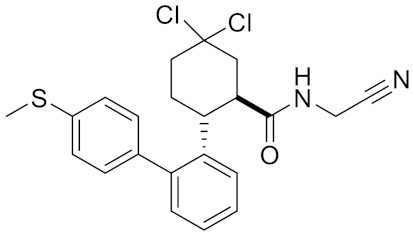	Inhibitor, IC50 = 0.58	[183]
Compound rac-38a	C22H23FN2OS	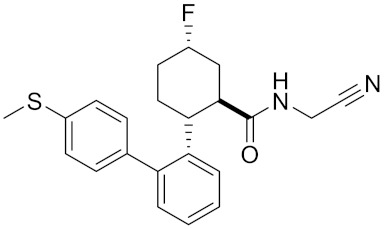	Inhibitor, IC50 = 4.2	[183]
Compound rac-38b	C22H23FN2OS	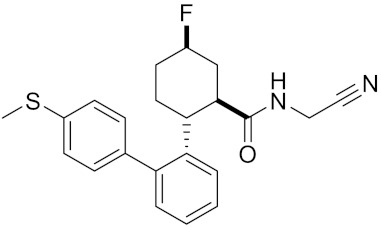	Inhibitor, IC50 = 3.7	[183]
Compound 1a		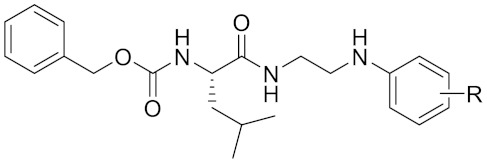	R = H, Inhibitor, IC50 = 0.47	[178]
Compound 1b			R = 3-CH3, Inhibitor, IC50 = 0.46	
Compound 1c			R = 4-CH3, Inhibitor, IC50 = 0.19	
Compound 1d			R = 3-Cl, Inhibitor, IC50 > 1	
Compound 1e			R = 4-Cl, Inhibitor, IC50 = 0.35	
Compound 1f			R = 3-OCH3, Inhibitor, IC50 > 1	
Compound 1g			R = 4-OCH3, Inhibitor, IC50 = 0.06	
Tri-Ring P3 Benzamide-Containing Aminonitriles		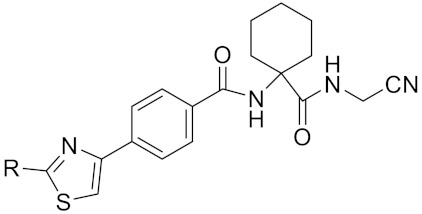	Inhibitor, Ki < 0.003 nM	[180]
		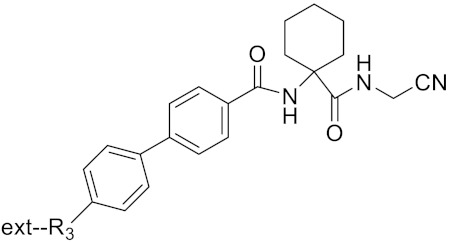	Inhibitor, Ki < 0.00025 nM	
Nonpeptidic Cyanamides		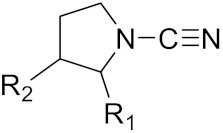	Inhibitor, IC50 = 0.05–13.7 μM	[142]
Compound 4a		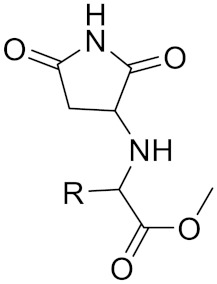	R= GlyOMe, Inhibitor, IC50 = 0.1 mM	[184]
Compound 4d			R= L-AsnOMe, Inhibitor, IC50 = 0.4 mM	[184]
Amentoflavone		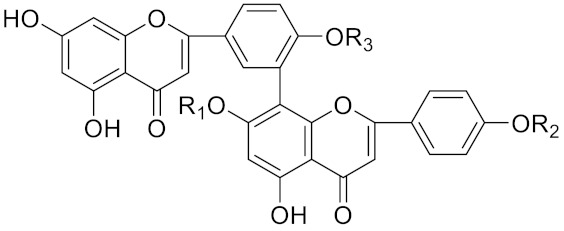	R1 = R2 = R3 = H, Inhibitor, IC50 = 1.88 μM	[185]
Podocarpusflavone A,			R1= R3 = H, R2 = CH3, Inhibitor, IC50 = 2.51 μM	[185]
7′′,4′′′-dimethylamentoflavone			R1= R2 = CH3, R3 = H, Inhibitor, IC50 = 1.57 μM	[185]
Bilobetin			R1= R2 = H, R3 = CH3, Inhibitor, IC50 = 1.55 μM	[185]
2,3-dihydroamentoflavone	C30H18O10	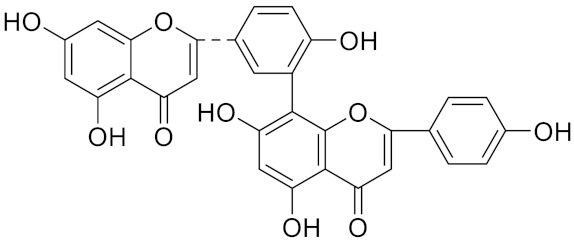	Inhibitor, IC50 = 1.39 μM	[185]
Hinokiflavone	C30H18O10	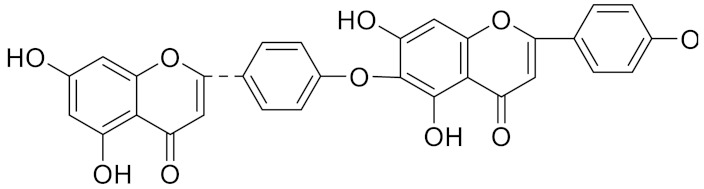	Inhibitor, IC50 = 8.797 μM	[185]
Kushennol F	C25H28O6	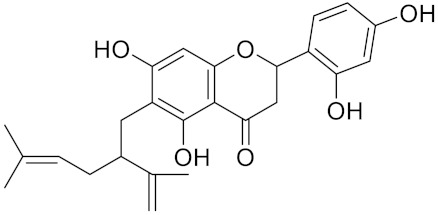	Inhibitor, IC50 = 27.24 nM	[186]
Sophoraflavone G	C25H28O6	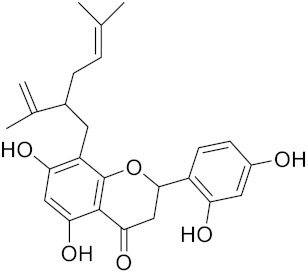	Inhibitor, IC50 = 1.54 nM	[186]
A series ofketoamides with varying P1 moieties		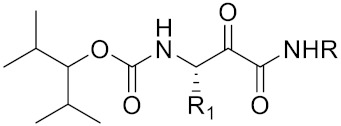	Inhibitor, IC50 = 0.77–12,000 nM	[187]

## 12. Conclusions

CTSK is expressed in several tissue carcinomas, including breast, prostate, lung, kidney, colon, stomach and ovarian cancers. It plays an important role in promoting the invasion and migration of cancer cells. The CTSK levels are higher than normal after cancer metastasis, and this change might be used to diagnose and assess the severity of cancer patients. Based on the role of CTSK in a variety of cancers, it may serve as a potential biomarker.

The inhibition of CTSK attenuates osteolytic lesions and, thus, inhibits cancer progression. Activating CTS promoted RANKL-induced osteoclast formation and osteoclast-mediated bone resorption activity. CTSK inhibitors have been studied clinically. They were able to significantly reduce the biochemical indicators of bone resorption, serum CTX and urinary NTX levels and increase the bone mineral density.

In conclusion, in this review, we summarized that CTSK may be a novel cancer biomarker and therapeutic target. In order to verify that CTSK is more specific in the treatment of cancers and seek better treatment results, a large number of studies and more in-depth mechanism studies are still needed.

## Figures and Tables

**Table 1:** The effects and mechanisms of CTSK in cancers.

Cancer Type	Research Samples	Effects	Mechanism	Refs.
Prostate				
Prostate cancer	LNCaP cells; C4-2B cells; PC3 cells; Patient tissue; Patient serum	CTSK promotes invasion and metastasis of prostate cancer; CTSK promotes cytokine Release;	CTSK mediates bone matrix degradation; CTSK is involved in CCL2- and Cox-2-driven pathways;	[44]
**Breast**				
Breast cancer	MCF-7 cells; MDA-MB-231 cells; Hs578T cells	CTSK is highly expressed in mammary fibroblasts; CTSK promotes breast cancer cells to metastasize to other sites	CTSK interacts with RANKL/RANK; CTSK degrades ECM and activates MMP-9 to promote breast cancer metastasis; CTSK promoter selectively overexpresses BCA3, which interacts with Rac1 and is associated with NF-κB signaling.	[21,54,127,128]
Breast cancer with bone metastasis	MDA-MB-231Hi cells	CTSK is strongly expressed after bone metastases in breast cancer; CTSK degrade extracellular matrix;	Both RANKL and TGF-β can induce the transcription factor NFATc1 to accumulate in the nucleus, and NFATc1 binds to the promoter and directly induces the expression of Src and CTSK; The high expression of IL-20 upregulates CTSK and MMP-9, and promotes bone metastasis of breast cancer	[66]
**Bone**				
Giant cell tumor of bone	osteoclast-like giant cells; multinucleated giant cells	CTSK is abundantly expressed in the multinucleated giant cells and its activity, which was more than 100-fold higher than activities found in other tissues expressing CTSK	CTSK degrades collagen matrix; CTSK/V-ATPase system is primarily proteolytic factors leading to osteolysis of GCT of bone	[18]
**Kidney**				
CRC	Xp11TRC cells; Renal cells	CTSK is positive; CTSK can be used as an immunodiagnostic marker	CTSK is a transcriptional target of Mitf and TFE3; TFE3 gene rearrangement and TFEB gene amplification lead to the invasion and migration of renal cell carcinoma, and the expression level of CTSK is higher; t (6;11) (p21; q12) translocation; TSC1/TSC2 mutation promotes renal cell carcinoma development; CTSK inhibitor significantly reduced mTOR phosphorylation at S2448 in Caki cells and inhibited renal cell carcinoma progression.	[76,77,78,119,122]
CCRCC	CCRCC cells	CTSK is positive	CTSK and mTOR expression is dysregulated	[81,82]
PRCC	PRCC cells	CTSK is positive	CTSK and mTOR expression is dysregulated	[28]
CRCC	CRCC cells	CTSK is positive	CTSK and mTOR expression is dysregulated	[28]
Sporadic RCC	RCC cells	CTSK is positive	CTSK and mTOR expression is dysregulated	[28,123]
**Lung**				
NSCLC	SBC-5 cells; A549 cells; Patient tissues; Patient serum; Mice tissues	CTSK maintains airway structural integrity; CTSK strongly expressed;	CTSK binds to TGF-β1 and activates the pathway to promote tumor metastasis; CTSK promotes NSCLC invasion and metastasis by activating mTOR signaling pathway; mTOR/S6K/rS6; EGFR/Akt/mTOR pathway plays a role;	[129,130,131,132,133]
ADC	Patient tissues	CTSK is positive	TGF-β1 acts as a potent substrate for CTSK.	[133]
ADC	Patient tissues	CTSK is positive	CTSK activates mTOR signaling pathway mTOR/S6K/rS6 axis comes into play;	[134]
SqCC	Patient tissues	CTSK is positive	Increased expression of p-mTOR, so it associated with the mTOR pathway	[133,135]
LCC	Patient tissues	Not mentioned	Not mentioned	
**Others**				
Colorectal cancer	MC38 cells; SW480 cells; RKO cells; Mice serum	Serum CTSK levels were significantly elevated in mice with intestinal flora imbalance; CTSK contributes to the aggressive phenotype of CRC cells in vitro and in vivo; CTSK as a prognostic biomarker	APC, TP53, KRAS, SMAD4 and PIK3CA mutations promote the occurrence of colorectal cancer; CTSK can stimulate M2-TAMs to secrete cytokines such as IL-10 and IL-17, and then promote the invasion and metastasis of colorectal cancer cells through the NF-κB pathway; CTSK binds to TLR4 and stimulates M2 polarization in tumor-associated macrophages through mTOR pathway. TLR signaling leads to phosphorylation and activation of microphthalmia transcription factors through p38, acting on the CTSK promoter	[94,95,97,99]
Ovarian cancer	OCa cells; Patient serum	CTSK promotes the metastasis of ovarian cancer; The expression of CTSK in peritoneal metastatic ovarian cancer was significantly higher than that in primary ovarian cancer; CTSK is a useful marker for the diagnosis of primary OCs with specific enhancement in combination with CA25 and HE4	AGAP2-AS1 inhibits cell metastasis and proliferation by inhibiting epithelial-mesenchymal transition by downregulating CTSK; Downregulation of NDRG1 reduces the expression of pro-invasive genes CTSK, MMP-7 and TMPRSS4	[101,104,105,106]
Gastric cancer	AGS cells; Gastric epithelial cells and macrophages; Gastric parietal cells	high expression of CTSK in gastric cancer cells; CTSK promotes the metastasis of gastric cancer;	CTSK promotes gastric cancer metastasis as a downstream factor of cytoskeletal protein Coronin 3; CTSK rapidly degrades ECM; CTSK cleaves and activates MMP-9 to promote gastric cancer cell proliferation and migration;	[108]
Melanoma	Melanoma cells	CTSK is significantly expressed in skin and fibroblasts; CTSK is positive in most primary melanomas and all cutaneous melanoma metastases;	Through the secretion of MMP and CTSK through the lymph and blood, the internal collagen is cut off, which promotes the penetration of melanoma cells into the dermis and achieves distant metastasis; CTSK may mediate the degradation of matrix proteins after phagocytosis and promote the invasion and metastasis of melanocytes	[113,114,115]

## References

[1] Torre L.A., Islami F., Siegel R.L., Ward E.M., Jemal A. (2017). Global cancer in women: Burden and trends. Cancer Epidemiol. Biomark. Prev..

[2] Mattiuzzi C., Lippi G. (2019). Current cancer epidemiology. J. Epidemiol. Glob. Health.

[3] Tompkins K.D., Thorburn A. (2019). Regulation of apoptosis by autophagy to enhance cancer therapy. Yale J. Biol. Med..

[4] Neville B.W., Day T.A. (2002). Oral cancer and precancerous lesions. CA Cancer J. Clin..

[5] Rossi A., Deveraux Q., Turk B., Sali A. (2004). Comprehensive search for cysteine cathepsins in the human genome. Biol. Chem..

[6] Abdel-Magid A.F. (2015). Inhibition of cathepsin K: A novel and promising treatment for osteoporosis. ACS Med. Chem. Lett..

[7] Drake F.H., Dodds R.A., James I.E., Coleman L., Rieman D., Barthlow R., Lee-Rykaczewski E., Coleman L., Rieman D., Barthlow R. (1996). Cathepsin K, but not cathepsins B, L, or S, is abundantly expressed in human osteoclasts. J. Biol. Chem..

[8] Yang J., Shang G.D., Zhang Y.Q. (2004). Study of a novel antiosteoporosis screening model targeted on cathepsin K. Biomed. Environ. Sci..

[9] Rünger T.M., Quintanilla-Dieck M.J., Bhawan J. (2007). Role of cathepsin K in the turnover of the dermal extracellular matrix during scar formation. J. Investig. Dermatol..

[10] Gelb B.D., Shi G.P., Chapman H.A., Desnick R.J. (1996). Pycnodysostosis, a lysosomal disease caused by cathepsin K deficiency. Science.

[11] Garnero P., Borel O., Byrjalsen I., Ferreras M., Drake F.H., McQueney M.S., Foged N.T., Delmas P.D., Delaissé J.M. (1998). The collagenolytic activity of cathepsin K is unique among mammalian proteinases. J. Biol. Chem..

[12] Cheng X. (2008). The expression and significance of collagenolyitc cathepsin K in the left ventricle remodeling during the hypertensive heart failure. Clin. Med. J. Chin..

[13] Dai Q., Xie F., Han Y., Ma X., Zhou S., Jiang L., Zou W., Wang J. (2017). Inactivation of regulatory-associated protein of mTOR (raptor)/mammalian target of rapamycin complex 1 (mTORC1) signaling in osteoclasts increases bone mass by inhibiting osteoclast differentiation in mice. J. Biol. Chem..

[14] Li X., Li Y., Jin J., Jin D., Cui L., Li X., Rei Y., Jiang H., Zhao G., Yang G. (2014). Increased serum cathepsin K in patients with coronary artery disease. Yonsei Med. J..

[15] Hu L., Cheng X.W., Song H., Inoue A., Jiang H., Li X., Shi G.P., Kozawa E., Okumura K., Kuzuya M. (2014). Cathepsin K activity controls injury-related vascular repair in mice. Hypertension.

[16] Wang X., Liu D.J., Guo Y.Y., Zhang J.L., Zhang X.L. (2021). Role of cathepsin in rheumatoid arthritis. Pract. Pharm. Clin. Remed..

[17] Littlewood-Evans A.J., Bilbe G., Bowler W.B., Farley D., Wlodarski B., Kokubo T., Inaoka T., Sloane J., Evans D.B., Gallagher J.A. (1997). The osteoclast-associated protease cathepsin K is expressed in human breast carcinoma. Cancer Res..

[18] Lindeman J.H., Hanemaaijer R., Mulder A., Dijkstra P.D., Szuhai K., Bromme D., Verheijen J.H., Hogendoorn P.C. (2004). Cathepsin K is the principal protease in giant cell tumor of bone. Am. J. Pathol..

[19] Brubaker K.D., Vessella R.L., True L.D., Thomas R., Corey E. (2003). Cathepsin K mRNA and protein expression in prostate cancer progression. J. Bone Miner. Res..

[20] Joyce J.A., Baruch A., Chehade K., Meyer-Morse N., Giraudo E., Tsai F.Y., Greenbaum D.C., Hager J.H., Bogyo M., Hanahan D. (2004). Cathepsin cysteine proteases are effffectors of invasive growth and angiogenesis during multistage tumorigenesis. Cancer Cell.

[21] Kleer C.G., Bloushtain-Qimron N., Chen Y.H., Carrasco D., Hu M., Yao J., Kraeft S.K., Collins L.C., Sabel M.S., Argani P. (2008). Epithelial and stromal cathepsin K and CXCL14 expression in breast tumor progression. Clin. Cancer Res..

[22] Boutté A.M., Friedman D.B., Bogyo M., Min Y., Yang L., Lin P.C. (2011). Identifification of a myeloid-derived suppressor cell cystatin-like protein that inhibits metastasis. FASEB J..

[23] Rolli M., Fransvea E., Pilch J., Saven A., Felding-Habermann B. (2003). Activated integrin alphavbeta3 cooperates with metalloproteinase MMP-9 in regulating migration of metastatic breast cancer cells. Proc. Natl. Acad. Sci. USA.

[24] Hiratsuka S., Nakamura K., Iwai S., Murakami M., Itoh T., Kijima H., Shipley J.M., Senior R.M., Shibuya M. (2002). MMP9 induction by vascular endothelial growth factor receptor-1 is involved in lung-specific metastasis. Cancer Cell.

[25] Christensen J., Shastri V.P. (2015). Matrix-metalloproteinase-9 is cleaved and activated by cathepsin K. BMC Res. Notes.

[26] Parks A.N., Nahata J., Edouard N.E., Temenoff J.S., Platt M.O. (2019). Sequential, but not concurrent, incubation of cathepsin K and L with type I collagen results in extended proteolysis. Sci. Rep..

[27] Corisdeo S., Gyda M., Zaidi M., Moonga B.S., Troen B.R. (2001). New insights into the regulation of cathepsin K gene expression by osteoprotegerin ligand. Biochem. Biophys. Res. Commun..

[28] Podgorski I., Linebaugh B.E., Koblinski J.E., Rudy D.L., Herroon M.K., Olive M.B., Sloane B.F. (2009). Bone marrow-derived cathepsin K cleaves SPARC in bone metastasis. Am. J. Pathol..

[29] Terpos E., Confavreux C.B., Clézardin P. (2015). Bone antiresorptive agents in the treatment of bone metastases associated with solid tumours or multiple myeloma. Bonekey Rep..

[30] Hofbauer L.C., Rachner T.D., Coleman R.E., Jakob F. (2014). Endocrine aspects of bone metastases. Lancet Diabetes Endocrinol..

[31] Coleman R., Gnant M., Morgan G., Clezardin P. (2012). Effects of bone-targeted agents on cancer progression and mortality. J. Natl. Cancer Inst..

[32] Seo S.U., Woo S.M., Kim M.W., Lee H.S., Kim S.H., Kang S.C., Lee E.W., Min K.J., Kwon T.K. (2020). Cathepsin K inhibition-induced mitochondrial ROS enhances sensitivity of cancer cells to anti-cancer drugs through USP27x-mediated Bim protein stabilization. Redox Biol..

[33] Guri Y., Hall M.N. (2016). mTOR Signaling confers resistance to targeted cancer drugs. Trends Cancer.

[34] Kimura T., Egawa S. (2018). Epidemiology of prostate cancer in Asian countries. Int. J. Urol..

[35] Kohaar I., Petrovics G., Srivastava S. (2019). A rich array of prostate cancer molecular biomarkers: Opportunities and challenges. Int. J. Mol. Sci..

[36] Catalona W.J., Richie J.P., Ahmann F.R., Hudson M.A., Scardino P.T., Flanigan R.C., DeKernion J.B., Ratliff T.L., Kavoussi L.R., Dalkin B.L. (2017). Comparison of digital rectal examination and serum prostate specific antigen in the early detection of prostate cancer: Results of a multicenter clinical trial of 6630 men. J. Urol..

[37] Zhang X. (2019). Interactions between cancer cells and bone microenvironment promote bone metastasis in prostate cancer. Cancer Commun..

[38] Eguchi K., Akiba Y., Akiba N., Nagasawa M., Cooper L.F., Uoshima K. (2018). Insulin-like growth factor binding Protein-3 suppresses osteoblast differentiation via bone morphogenetic protein-2. Biochem. Biophys. Res. Commun..

[39] Salazar V.S., Gamer L.W., Rosen V. (2016). BMP signalling in skeletal development, disease and repair. Nat. Rev. Endocrinol..

[40] Verrecchia F., Rédini F. (2018). Transforming growth factor-β signaling plays a pivotal role in the interplay between osteosarcoma cells and their microenvironment. Front. Oncol..

[41] Florencio-Silva R., Sasso G.R., Sasso-Cerri E., Simões M.J., Cerri P.S. (2015). Biology of bone tissue: Structure, function, and factors that influence bone cells. Biomed. Res. Int..

[42] Burr D.B. (2019). Changes in bone matrix properties with aging. Bone.

[43] Vidak E., Javoršek U., Vizovišek M., Turk B. (2019). Cysteine cathepsins and their extracellular roles: Shaping the microenvironment. Cells.

[44] Akram M., Iqbal M., Daniyal M., Khan A.U. (2017). Awareness and current knowledge of breast cancer. Biol. Res..

[45] Samavat H., Kurzer M.S. (2015). Estrogen metabolism and breast cancer. Cancer Lett..

[46] Trabert B., Sherman M.E., Kannan N., Stanczyk F.Z. (2020). Progesterone and breast cancer. Endocr. Rev..

[47] Tian J.M., Ran B., Zhang C.L., Yan D.M., Li X.H. (2018). Estrogen and progesterone promote breast cancer cell proliferation by inducing cyclin G1 expression. Braz. J. Med. Biol. Res..

[48] Le Gall C., Bellahcène A., Bonnelye E., Gasser J.A., Castronovo V., Green J., Zimmermann J., Clézardin P. (2007). A cathepsin K inhibitor reduces breast cancer induced osteolysis and skeletal tumor burden. Cancer Res..

[49] Muschler J., Streuli C.H. (2010). Cell-matrix interactions in mammary gland development and breast cancer. Cold Spring Harb. Perspect. Biol..

[50] Lugo-Cintrón K.M., Gong M.M., Ayuso J.M., Tomko L.A., Beebe D.J., Virumbrales-Muñoz M., Ponik S.M. (2020). Breast fibroblasts and ECM components modulate breast cancer cell migration through the secretion of MMPs in a 3D microfluidic co-culture model. Cancers.

[51] Wu X., Li F., Dang L., Liang C., Lu A., Zhang G. (2020). RANKL/RANK system-based mechanism for breast cancer bone metastasis and related therapeutic strategies. Front. Cell Dev. Biol..

[52] Jones D.H., Nakashima T., Sanchez O.H., Kozieradzki I., Komarova S.V., Sarosi I., Morony S., Rubin E., Sarao R., Hojilla C.V. (2006). Regulation of cancer cell migration and bone metastasis by RANKL. Nature.

[53] Tu Q., Zhang J., Fix A., Brewer E., Li Y.P., Zhang Z.Y., Chen J. (2009). Targeted overexpression of BSP in osteoclasts promotes bone metastasis of breast cancer cells. J. Cell. Physiol..

[54] Guo Y., Tiedemann K., Khalil J.A., Russo C., Siegel P.M., Komarova S.V. (2008). Osteoclast precursors acquire sensitivity to breast cancer derived factors early in differentiation. Bone.

[55] Motyckova G., Weilbaecher K.N., Horstmann M., Rieman D.J., Fisher D.Z., Fisher D.E. (2001). Linking osteopetrosis and pycnodysostosis: Regulation of cathepsin K expression by the microphthalmia transcription factor family. Proc. Natl. Acad. Sci. USA.

[56] Montgomery N., Hill A., McFarlane S., Neisen J., O’Grady A., Conlon S., Jirstrom K., Kay E.W., Waugh D.J. (2012). CD44 enhances invasion of basal-like breast cancer cells by upregulating serine protease and collagen-degrading enzymatic expression and activity. Breast Cancer Res..

[57] Hsu Y.H., Hsing C.H., Li C.F., Chan C.H., Chang M.C., Yan J.J., Chang M.S. (2012). Anti-IL-20 monoclonal antibody suppresses breast cancer progression and bone osteolysis in murine models. J. Immunol..

[58] Gruenwald N., Demos T.C., Lomasney L.M., Rapp T. (2006). Giant-cell tumor. Orthopedics.

[59] Wülling M., Engels C., Jesse N., Werner M., Delling G., Kaiser E. (2001). The nature of giant cell tumor of bone. J. Cancer Res. Clin. Oncol.

[60] Kiviranta R., Morko J., Uusitalo H., Aro H.T., Vuorio E., Rantakokko J. (2001). Accelerated turnover of metaphyseal trabecular bone in mice overexpressing cathepsin K. J. Bone Miner. Res..

[61] Gowen M., Lazner F., Dodds R., Kapadia R., Feild J., Tavaria M., Bertoncello I., Drake F., Zavarselk S., Tellis I. (1999). Cathepsin K knockout mice develop osteopetrosis due to a deficit in matrix degradation but not demineralization. J. Bone Miner. Res..

[62] Van der Heijden L., Dijkstra P.D., van de Sande M.A., Kroep J.R., Nout R.A., van Rijswijk C.S., Bovée J.V., Hogendoorn P.C., Gelderblom H. (2014). The clinical approach toward giant cell tumor of bone. Oncologist.

[63] Liao T.S., Yurgelun M.B., Chang S.S., Zhang H.Z., Murakami K., Blaine T.A., Parisien M.V., Kim W., Winchester R.J., Lee F.Y. (2005). Recruitment of osteoclast precursors by stromal cell derived factor-1 (SDF-1) in giant cell tumor of bone. J. Orthop. Res..

[64] Branstetter D.G., Nelson S.D., Manivel J.C., Blay J.Y., Chawla S., Thomas D.M., Jun S., Jacobs I. (2012). Denosumab induces tumor reduction and bone formation in patients with giant-cell tumor of bone. Clin. Cancer Res..

[65] Chawla S., Henshaw R., Seeger L., Choy E., Blay J.Y., Ferrari S., Kroep J., Grimer R., Reichardt P., Rutkowski P. (2013). Safety and efficacy of denosumab for adults and skeletally mature adolescents with giant cell tumour of bone: Interim analysis of an open-label, parallel-group, phase 2 study. Lancet Oncol..

[66] Murai M., Oya M. (2004). Renal cell carcinoma: Etiology, incidence and epidemiology. Curr. Opin. Urol..

[67] Jonasch E., Gao J., Rathmell W.K. (2014). Renal cell carcinoma. BMJ.

[68] Akgul M., Williamson S.R., Ertoy D., Argani P., Gupta S., Caliò A., Reuter V., Tickoo S., Al-Ahmadie H.A., Netto G.J. (2021). Diagnostic approach in TFE3-rearranged renal cell carcinoma: A multi-institutional international survey. J. Clin. Pathol..

[69] Martignoni G., Gobbo S., Camparo P., Brunelli M., Munari E., Segala D., Pea M., Bonetti F., Illei P.B., Netto G.J. (2011). Differential expression of cathepsin K in neoplasms harboring TFE3 gene fusions. Mod. Pathol..

[70] Martignoni G., Pea M., Gobbo S., Brunelli M., Bonetti F., Segala D., Pan C.C., Netto G., Doglioni C., Hes O. (2009). Cathepsin-K immunoreactivity distinguishes MiTF/TFE family renal translocation carcinomas from other renal carcinomas. Mod. Pathol..

[71] Wu H., He D., Biswas S., Shafiquzzaman M., Zhou X., Charron J., Wang Y., Nayak B.K., Habib S.L., Liu H. (2021). mTOR activation initiates renal cell carcinoma development by coordinating ERK and p38MAPK. Cancer Res..

[72] Cho D.C., Mier J.W. (2013). Dual inhibition of PI3-kinase and mTOR in renal cell carcinoma. Curr. Cancer Drug Targets.

[73] Coinu A., Petrelli F., Barni S. (2016). Optimal treatment of poor-risk renal cell carcinoma patients with mTOR inhibitors and anti-VEGFR agents. Expert Rev. Anticancer Ther..

[74] Iakymenko O.A., Delma K.S., Jorda M., Kryvenko O.N. (2021). Cathepsin K (clone EPR19992) demonstrates uniformly positive immunoreactivity in renal oncocytoma, chromophobe renal cell carcinoma, and distal tubules. Int J. Surg. Pathol..

[75] Xia Q.Y., Wang X., Wei X., Wang X.T., Ma H.H., Lu Z.F., Rao Q. (2019). Eosinophilic solid and cystic renal cell carcinoma: Clinicopathological analysis and molecular characterization. Zhonghua Bing Li Xue Za Zhi.

[76] Chen Y.B., Mirsadraei L., Jayakumaran G., Al-Ahmadie H.A., Fine S.W., Gopalan A., Sirintrapun S.J., Tickoo S.K., Reuter V.E. (2019). Somatic mutations of TSC2 or MTOR characterize a morphologically distinct subset of sporadic renal cell carcinoma with eosinophilic and vacuolated cytoplasm. Am. J. Surg. Pathol..

[77] Farcaş M., Gatalica Z., Trpkov K., Swensen J., Zhou M., Alaghehbandan R., Williamson S.R., Magi-Galluzzi C., Gill A.J., Tretiakova M. (2022). Eosinophilic vacuolated tumor (EVT) of kidney demonstrates sporadic TSC/MTOR mutations: Next-generation sequencing multi-institutional study of 19 cases. Mod. Pathol..

[78] McDorman K.S., Wolf D.C. (2002). Use of the spontaneous TSC2 knockout (Eker) rat model of hereditary renal cell carcinoma for the study of renal carcinogens. Toxicol. Pathol..

[79] Sahin K., Cross B., Sahin N., Ciccone K., Suleiman S., Osunkoya A.O., Master V., Harris W., Carthon B., Mohammad R. (2015). Lycopene in the prevention of renal cell cancer in the TSC2 mutant Eker rat model. Arch. Biochem. Biophys..

[80] Palsgrove D.N., Li Y., Pratilas C.A., Lin M.T., Pallavajjalla A., Gocke C., de Marzo A.M., Matoso A., Netto G.J., Epstein J.I. (2018). Eosinophilic solid and cystic (ESC) renal cell carcinomas harbor TSC mutations: Molecular analysis supports an expanding clinicopathologic spectrum. Am. J. Surg. Pathol..

[81] Tretiakova M.S. (2018). Eosinophilic solid and cystic renal cell carcinoma mimicking epithelioid angiomyolipoma: Series of 4 primary tumors and 2 metastases. Hum. Pathol..

[82] Martignoni G., Bonetti F., Chilosi M., Brunelli M., Segala D., Amin M.B., Argani P., Eble J.N., Gobbo S., Pea M. (2012). Cathepsin K expression in the spectrum of perivascular epithelioid cell (PEC) lesions of the kidney. Mod. Pathol..

[83] Wang J., You H., Qi J., Yang C., Ren Y., Cheng H. (2017). Autocrine and paracrine STIP1 signaling promote osteolytic bone metastasis in renal cell carcinoma. Oncotarget.

[84] Siegel R.L., Miller K.D., Jemal A. (2019). Cancer statistics, 2019. CA Cancer J. Clin..

[85] Travis W.D., Brambilla E., Yatabe Y., Austin J.H.M., Beasley M.B., Chirieac L.R., Dacic S., Duhig E., Flieder D.B., WHO Panel (2015). The 2015 World Health Organization classification of lung tumors: Impact of genetic, clinical and radiologic advances since the 2004 classification. J. Thorac. Oncol..

[86] Caliò A., Mengoli M.C., Cavazza A., Rossi G., Ghimenton C., Brunelli M., Pea M., Chilosi M., Marcolini L., Martignoni G. (2018). Cathepsin K expression in clear cell “sugar” tumor (PEComa) of the lung. Virchows Arch..

[87] Bühling F., Peitz U., Wex T., Küster D., Vieth M., Gebert I., Roessner A., Weber E., Malfertheiner P., Wex T. (2004). Cathepsins K, L, B, X and W are differentially expressed in normal and chronically inflamed gastric mucosa. Biol. Chem..

[88] Zhang D., Leung N., Weber E., Saftig P., Brömme D. (2011). The effect of cathepsin K deficiency on airway development and TGF-β1 degradation. Respir. Res..

[89] Li M., Amizuka N., Takeuchi K., Freitas P.H., Kawano Y., Hoshino M., Oda K., Nozawa-Inoue K., Maeda T. (2006). Histochemical evidence of osteoclastic degradation of extracellular matrix in osteolytic metastasis originating from human lung small carcinoma (SBC-5) cells. Microsc. Res. Tech..

[90] Naumnik W., Niklińska W., Ossolińska M., Chyczewska E. (2009). Serum cathepsin K and cystatin C concentration in patients with advanced non-small-cell lung cancer during chemotherapy. Folia Histochem. CytoBiol..

[91] Wang R., Zhang J., Chen S., Lu M., Luo X., Yao S., Liu S., Qin Y., Chen H. (2011). Tumor-associated macrophages provide a suitable microenvironment for non-small lung cancer invasion and progression. Lung Cancer.

[92] Yang H., Heyer J., Zhao H., Liang S., Guo R., Zhong L. (2020). The Potential Role of Cathepsin K in Non-Small Cell Lung Cancer. Molecules.

[93] Belhamidi M.S., Sinaa M., Kaoukabi A., Krimou H., Menfaa M., Sakit F., Choho A. (2018). Profil épidémiologique et anatomopathologique du cancer colorectal: À propos de 36 caswe [Epidemiological and pathological profile of colorectal cancer: About 36 cases]. Pan Afr. Med. J..

[94] Adeleke S., Haslam A., Choy A., Diaz-Cano S., Galante J.R., Mikropoulos C., Boussios S. (2022). Microsatellite instability testing in colorectal patients with Lynch syndrome: Lessons learned from a case report and how to avoid such pitfalls. Pers. Med..

[95] Müller M.F., Ibrahim A.E., Arends M.J. (2016). Molecular pathological classification of colorectal cancer. Virchows Arch..

[96] Li R., Zhou R., Wang H., Li W., Pan M., Yao X., Zhan W., Yang S., Xu L., Ding Y. (2019). Gut microbiota-stimulated cathepsin K secretion mediates TLR4-dependent M2 macrophage polarization and promotes tumor metastasis in colorectal cancer. Cell Death Differ..

[97] Arthur J.C., Perez-Chanona E., Mühlbauer M., Tomkovich S., Uronis J.M., Fan T.J., Campbell B.J., Abujamel T., Dogan B., Rogers A.B. (2012). Intestinal inflammation targets cancer-inducing activity of the microbiota. Science.

[98] Hurtado C.G., Wan F., Housseau F., Sears C.L. (2018). Roles for interleukin 17 and adaptive immunity in pathogenesis of colorectal cancer. Gastroenterology.

[99] Li L., Zhu Z., Zhao Y., Zhang Q., Wu X., Miao B., Cao J., Fei S. (2019). FN1, SPARC, and SERPINE1 are highly expressed and significantly related to a poor prognosis of gastric adenocarcinoma revealed by microarray and bioinformatics. Sci. Rep..

[100] Makondi P.T., Wei P.L., Huang C.Y., Chang Y.J. (2019). Development of novel predictive miRNA/target gene pathways for colorectal cancer distance metastasis to the liver using a bioinformatic approach. PLoS ONE.

[101] Gaona-Luviano P., Medina-Gaona L.A., Magaña-Pérez K. (2020). Epidemiology of ovarian cancer. Chin. ClinOncol.

[102] Cho K.R., Shih I.-M. (2009). Ovarian cancer. Annu. Rev. Pathol..

[103] Xu H., Ma Y., Lu B., Pan Z., Lu Y., Liu P., Lu B. (2016). Identification of cathepsin K in the peritoneal metastasis of ovarian carcinoma using in-silico, gene expression analysis. J. Cancer.

[104] Tingting Z., Xiaojing L., Junjun Q., Keqin H., Junjun Q. (2020). The antisense long noncoding RNA AGAP2-AS1 regulates cell proliferation and metastasis in epithelial ovarian cancer. J. Cancer.

[105] Zhao G., Chen J., Zhao Z., Gao F., Zhu J., Feng Z., Lv X., Zhao Z. (2011). Identification of NDRG1-regulated genes associated with invasive potential in cervical and ovarian cancer cells. Biochem. Biophys. Res. Commun..

[106] Weiland F., Martin K., Oehler M.K., Hoffmann P. (2012). Deciphering the molecular nature of ovarian cancer biomarker CA125. Int. J. Mol. Sci..

[107] Ang T.L., Fock K.M. (2014). Clinical epidemiology of gastric cancer. Singap. Med. J..

[108] Ren G., Tian Q., Fan D., Feng B., Lu Y., Liang J., Li K., Shang Y., Nie Y., Wang X. (2012). Coronin 3 promotes gastric cancer metastasis via the up-regulation of MMP-9 and cathepsin K. Mol. Cancer.

[109] Digklia A., Wagner A.D. (2016). Advanced gastric cancer: Current treatment landscape and future perspectives. World J. Gastroenterol..

[110] Slominski A., Wortsman J., Carlson A.J., Matsuoka L.Y., Balch C.M., Mihm M.C. (2001). Malignant melanoma. Arch. Pathol. Lab. Med.

[111] Sánchez-Danés A., Blanpain C. (2018). Deciphering the cells of origin of squamous cell carcinomas. Nat. Rev. Cancer.

[112] Boussios S., Rassy E., Samartzis E., Moschetta M., Sheriff M., Pérez-Fidalgo J.A., Pavlidis N. (2021). Melanoma of unknown primary: New perspectives for an old story. Crit. Rev. Oncol. Hematol..

[113] Quintanilla-Dieck M.J., Codriansky K., Keady M., Bhawan J., Rünger T.M. (2009). Expression and regulation of cathepsin K in skin fibroblasts. Exp. Dermatol..

[114] Quintanilla-Dieck M.J., Codriansky K., Rünger T.M., Bhawan J., Rünger T.M. (2008). Cathepsin K in melanoma invasion. J. Investig. Dermatol..

[115] Petricevic S.J., Pavlovic A., Durdov M.G., Becic K., Durdov M.G. (2017). Cathepsin K expression in melanoma is associated with metastases. Histol. Histopathol..

[116] Rao Q., Wang Y., Xia Q.Y., Shi S.S., Shen Q., Tu P., Shi Q.L., Zhou X.J., Wu B. (2014). Cathepsin K in the immunohistochemical diagnosis of melanocytic lesions. Int. J. Clin. Exp. Pathol..

[117] Yao C., Yu K.P., Philbrick W., Sun B.H., Simpson C., Zhang C., Insogna K. (2017). Breast cancer-associated gene 3 interacts with Rac1 and augments NF-κB signaling in vitro, but has no effect on RANKL-induced bone resorption in vivo. Int. J. Mol. Med..

[118] Jensen A.B., Wynne C., Ramirez G., He W., Song Y., Berd Y., Wang H., Mehta A., Lombardi A. (2010). The cathepsin K inhibitor odanacatib suppresses bone resorption in women with breast cancer and established bone metastases: Results of a 4-week, double-blind, randomized, controlled trial. Clin. Breast Cancer.

[119] Argani P., Hicks J., De Marzo A.M., Albadine R., Illei P.B., Ladanyi M., Reuter V.E., Netto G.J. (2010). Xp11 translocation renal cell carcinoma (RCC): Extended immunohistochemical profile emphasizing novel RCC markers. Am. J. Surg. Pathol..

[120] Mehner C., Hockla A., Miller E., Ran S., Radisky D.C., Radisky E.S. (2014). Tumor cell-produced matrix metalloproteinase 9 (MMP-9) drives malignant progression and metastasis of basal-like triple negative breast cancer. Oncotarget.

[121] Huang H. (2018). Matrix Metalloproteinase-9 (MMP-9) as a cancer biomarker and MMP-9 biosensors: Recent advances. Sensors.

[122] Guzińska-Ustymowicz K. (2006). MMP-9 and cathepsin B expression in tumor budding as an indicator of a more aggressive phenotype of colorectal cancer (CRC). Anticancer Res..

[123] Ahir B.K., Elias N.M., Lakka S.S. (2017). SPARC overexpression alters microRNA expression profiles involved in tumor progression. Genes Cancer.

[124] Shao H., Kirkwood J.M., Wells A. (2015). Tenascin-C signaling in melanoma. Cell Adhes. Migr..

[125] Yoshida T., Akatsuka T., Imanaka-Yoshida K. (2015). Tenascin-C and integrins in cancer. Cell Adhes. Migr..

[126] Spenlé C., Saupe F., Midwood K., Burckel H., Noel G., Orend G. (2015). Tenascin-C: Exploitation and collateral damage in cancer management. Cell Adhes. Migr..

[127] Kim H., Kim J., Lee S.K., Cho E.Y., Cho S.Y. (2019). TFE3-expressing perivascular epithelioid cell tumor of the breast. J. Pathol. Transl. Med..

[128] Tan M., Wu A., Liao N., Liu M., Guo Q., Yi J., Wang T., Huang Y., Qiu B., Zhou W. (2018). Inhibiting ROS-TFE3-dependent autophagy enhances the therapeutic response to metformin in breast cancer. Free Radic. Res..

[129] Bühling F., Röcken C., Brasch F., Hartig R., Yasuda Y., Saftig P., Brömme D., Welte T. (2004). Pivotal role of cathepsin K in lung fibrosis. Am. J. Pathol..

[130] Dobashi Y., Suzuki S., Matsubara H., Kimura M., Endo S., Ooi A. (2009). Critical and diverse involvement of Akt/mammalian target of rapamycin signaling in human lung carcinomas. Cancer.

[131] Dobashi Y., Suzuki S., Kimura M., Matsubara H., Tsubochi H., Imoto I., Ooi A. (2011). Paradigm of kinase-driven pathway downstream of epidermal growth factor receptor/Akt in human lung carcinomas. Hum. Pathol..

[132] Cordes C., Bartling B., Simm A., Afar D., Lautenschläger C., Hansen G., Silber R.E., Burdach S., Hofmann H.S. (2009). Simultaneous expression of Cathepsins B and K in pulmonary adenocarcinomas and squamous cell carcinomas predicts poor recurrence-free and overall survival. Lung Cancer.

[133] Hiramatsu M., Ninomiya H., Inamura K., Nomura K., Takeuchi K., Satoh Y., Okumura S., Nakagawa K., Yamori T., Matsuura M. (2010). Activation status of receptor tyrosine kinase downstream pathways in primary lung adenocarcinoma with reference of KRAS and EGFR mutations. Lung Cancer.

[134] Denisenko T.V., Budkevich I.N., Zhivotovsky B. (2018). Cell death-based treatment of lung adenocarcinoma. Cell Death Dis..

[135] Bühling F., Gerber A., Häckel C., Krüger S., Köhnlein T., Brömme D., Reinhold D., Ansorge S., Welte T. (1999). Expression of cathepsin K in lung epithelial cells. Am. J. Respir. Cell Mol. Biol..

[136] Duan Y., Tan Z., Yang M., Li J., Liu C., Wang C., Zhang F., Jin Y., Wang Y., Zhu L. (2019). PC-3-Derived Exosomes Inhibit Osteoclast Differentiation by Downregulating miR-214 and Blocking NF-κB Signaling Pathway. Biomed. Res. Int..

[137] Raimondi L., De Luca A., Amodio N., Manno M., Raccosta S., Taverna S., Bellavia D., Naselli F., Fontana S., Schillaci O. (2015). Involvement of multiple myeloma cell-derived exosomes in osteoclast differentiation. Oncotarget.

[138] Li H., Xiao Z., Quarles L.D., Li W. (2021). Osteoporosis: Mechanism, molecular target and current status on drug development. Curr. Med. Chem..

[139] Boonen S., Rosenberg E., Claessens F., Vanderschueren D., Papapoulos S. (2012). Inhibition of cathepsin K for treatment of osteoporosis. Curr. Osteoporos. Rep..

[140] Palermo C., Joyce J.A. (2008). Cysteine cathepsin proteases as pharmacological targets in cancer. Trends Pharmacol. Sci..

[141] Wang B., Li J., Ye Z., Wu X. (2014). N-myc downstream regulated gene 1 acts as a tumor suppressor in ovarian cancer. Oncol. Rep..

[142] Falgueyret J.P., Oballa R.M., Okamoto O., Wesolowski G., Aubin Y., Rydzewski R.M., Prasit P., Riendeau D., Rodan S.B., Percival M.D. (2001). Novel, nonpeptidic cyanamides as potent and reversible inhibitors of human cathepsins K and L. J. Med. Chem..

[143] Grabowskal U., Chambers T.J., Shiroo M. (2005). Recent developments in cathepsin K inhibitor design. Curr. Opin. Drug Discov. Dev..

[144] Vashum Y., Kottaiswamy A., Bupesh G., Singh R.P., Kalaiselvan P., Thiagarajan K., Samuel S. (2021). Inhibitory Effects of Cathepsin K Inhibitor (ODN-MK-0822) on the Paracrine Pro-Osteoclast Factors of Breast Cancer Cells. Curr. Mol. Pharmacol..

[145] Duong L.T., Wesolowski G.A., Leung P., Oballa R., Pickarski M. (2014). Efficacy of a cathepsin K inhibitor in a preclinical model for prevention and treatment of breast cancer bone metastasis. Mol. Cancer Ther..

[146] Liang W., Wang F., Chen Q., Dai J., Escara-Wilke J., Keller E.T., Zimmermann J., Hong N., Lu Y., Zhang J. (2019). Targeting cathepsin K diminishes prostate cancer establishment and growth in murine bone. J. Cancer Res. Clin. Oncol..

[147] Rapa I., Volante M., Cappia S., Rosas R., Scagliotti G.V., Papotti M. (2006). Cathepsin K is selectively expressed in the stroma of lung adenocarcinoma but not in bronchioloalveolar carcinoma: A useful marker of invasive growth. Am. J. Clin. Pathol..

[148] Stoch S.A., Zajic S., Stone J.A., Miller D.L., van Bortel L., Lasseter K.C., Pramanik B., Cilissen C., Liu Q., Liu L. (2013). Odanacatib, a selective cathepsin K inhibitor to treat osteoporosis: Safety, tolerability, pharmacokinetics and pharmacodynamics—Results from single oral dose studies in healthy volunteers. Br. J. Clin. Pharmacol..

[149] Kumar S., Dare L., Vasko-Moser J.A., James I.E., Blake S.M., Rickard D.J., Hwang S.M., Tomaszek T., Yamashita D.S., Marquis R.W. (2007). A highly potent inhibitor of cathepsin K (relacatib) reduces biomarkers of bone resorption both in vitro and in an acute model of elevated bone turnover in vivo in monkeys. Bone.

[150] Pennypacker B.L., Duong L.T., Cusick T.E., Masarachia P.J., Gentile M.A., Gauthier J.Y., Black W.C., Scott B.B., Samadfam R., Smith S.Y. (2011). Cathepsin K inhibitors prevent bone loss in estrogen-deficient rabbits. J. Bone Miner. Res..

[151] Jerome C., Missbach M., Gamse R. (2012). Balicatib, a cathepsin K inhibitor, stimulates periosteal bone formation in monkeys. Osteoporos. Int..

[152] Cusick T., Chen C.M., Pennypacker B.L., Pickarski M., Kimmel D.B., Scott B.B., Duong L.T. (2012). Odanacatib treatment increases hip bone mass and cortical thickness by preserving endocortical bone formation and stimulating periosteal bone formation in the ovariectomized adult rhesus monkey. J. Bone Miner. Res..

[153] Ochi Y., Yamada H., Mori H., Kawada N., Kayasuga R., Nakanishi Y., Tanaka M., Imagawa A., Ohmoto K., Kawabata K. (2014). ONO-5334, a cathepsin K inhibitor, improves bone strength by preferentially increasing cortical bone mass in ovariectomized rats. J. Bone Miner. Metab..

[154] Brixen K., Chapurlat R., Cheung A.M., Keaveny T.M., Fuerst T., Engelke K., Recker R., Dardzinski B., Verbruggen N., Ather S. (2013). Bone density, turnover, and estimated strength in postmenopausal women treated with odanacatib: A randomized trial. J. Clin. Endocrinol. Metab..

[155] Yi C., Hao K.Y., Ma T., Lin Y., Ge X.Y., Zhang Y. (2017). Inhibition of cathepsin K promotes osseointegration of titanium implants in ovariectomised rats. Sci. Rep..

[156] Kassahun K., McIntosh I., Koeplinger K., Sun L., Talaty J.E., Miller D.L., Dixon R., Zajic S., Stoch S.A. (2014). Disposition and metabolism of the cathepsin K inhibitor odanacatib in humans. Drug Metab. Dispos..

[157] Anderson M.S., Gendrano I.N., Liu C., Jeffers S., Mahon C., Mehta A., Mostoller K., Zajic S., Morris D., Lee J. (2014). Odanacatib, a selective cathepsin K inhibitor, demonstrates comparable pharmacodynamics and pharmacokinetics in older men and postmenopausal women. J. Clin. Endocrinol. Metab..

[158] Stone J.A., McCrea J.B., Witter R., Zajic S., Stoch S.A. (2019). Clinical and translational pharmacology of the cathepsin K inhibitor odanacatib studied for osteoporosis. Br. J. Clin. Pharmacol..

[159] Bonnick S., de Villiers T., Odio A., Palacios S., Chapurlat R., DaSilva C., Scott B.B., Le Bailly De Tilleghem C., Leung A.T., Gurner D. (2013). Effects of odanacatib on BMD and safety in the treatment of osteoporosis in postmenopausal women previously treated with alendronate: A randomized placebo-controlled trial. J. Clin. Endocrinol. Metab..

[160] Costa A.G., Cusano N.E., Silva B.C., Cremers S., Bilezikian J.P. (2011). Cathepsin K: Its skeletal actions and role as a therapeutic target in osteoporosis. Nat. Rev. Rheumatol..

[161] Dai R., Wu Z., Chu H.Y., Lu J., Lyu A., Liu J., Zhang G. (2020). Cathepsin K: The Action in and Beyond Bone. Front. Cell Dev. Biol..

[162] Hua Y., Xu X., Shi G.P., Chicco A.J., Ren J., Nair S. (2013). Cathepsin K knockout alleviates pressure overload-induced cardiac hypertrophy. Hypertension.

[163] Coluzzi F., Mandatori I., Mattia C. (2011). Emerging therapies in metastatic bone pain. Expert Opin. Emerg. Drugs.

[164] Gauthier J.Y., Chauret N., Cromlish W., Desmarais S., Duong L.T., Falgueyret J.P., Kimmel D.B., Lamontagne S., Léger S., LeRiche T. (2008). The discovery of odanacatib (MK-0822), a selective inhibitor of cathepsin K. Bioorg. Med. Chem. Lett..

[165] Leung P., Pickarski M., Zhuo Y., Masarachia P.J., Duong L.T. (2011). The effects of the cathepsin K inhibitor odanacatib on osteoclastic bone resorption and vesicular trafficking. Bone.

[166] Isabel E., Bateman K.P., Chauret N., Cromlish W., Desmarais S., Duong L.T., Falgueyret J.P., Gauthier J.Y., Lamontagne S., Lau C.K. (2010). The discovery of MK-0674, an orally bioavailable cathepsin K inhibitor. Bioorg. Med. Chem. Lett..

[167] Li C.S., Deschenes D., Desmarais S., Falgueyret J.P., Gauthier J.Y., Kimmel D.B., Léger S., Massé F., McGrath M.E., McKay D.J. (2006). Identification of a potent and selective non-basic cathepsin K inhibitor. Bioorg. Med. Chem. Lett..

[168] Zhuo Y., Gauthier J.Y., Black W.C., Percival M.D., Duong L.T. (2014). Inhibition of bone resorption by the cathepsin K inhibitor odanacatib is fully reversible. Bone.

[169] Desmarais S., Massé F., Percival M.D. (2009). Pharmacological inhibitors to identify roles of cathepsin K in cell-based studies: A comparison of available tools. Biol. Chem..

[170] Benýšek J., Buša M., Rubešová P., Fanfrlík J., Lepšík M., Brynda J., Matoušková Z., Bartz U., Horn M., Gütschow M. (2022). Highly potent inhibitors of cathepsin K with a differently positioned cyanohydrazide warhead: Structural analysis of binding mode to mature and zymogen-like enzymes. J. Enzym. Inhib. Med. Chem..

[171] Wu H., Yin G., Pu X., Wang J., Liao X., Huang Z. (2021). Inhibitory Effects of Combined Bone Morphogenetic Protein 2, Vascular Endothelial Growth Factor, and Basic Fibroblast Growth Factor on Osteoclast Differentiation and Activity. Tissue Eng. Part A.

[172] Asaad N., Bethel P.A., Coulson M.D., Dawson J.E., Ford S.J., Gerhardt S., Grist M., Hamlin G.A., James M.J., Jones E.V. (2009). Dipeptidyl nitrile inhibitors of Cathepsin L. Bioorg. Med. Chem. Lett..

[173] Yamashita D.S., Marquis R.W., Xie R., Nidamarthy S.D., Oh H.J., Jeong J.U., Erhard K.F., Ward K.W., Roethke T.J., Smith B.R. (2006). Structure activity relationships of 5-, 6-, and 7-methyl-substituted azepan-3-one cathepsin K inhibitors. J. Med. Chem..

[174] Pan W., Yin W., Yang L., Xue L., Ren J., Wei W., Lu Q., Ding H., Liu Z., Nabar N.R. (2019). Inhibition of Ctsk alleviates periodontitis and comorbid rheumatoid arthritis via downregulation of the TLR9 signalling pathway. J. Clin. Periodontol..

[175] Marquis R.W., Ru Y., LoCastro S.M., Zeng J., Yamashita D.S., Oh H.J., Erhard K.F., Davis L.D., Tomaszek T.A., Tew D. (2001). Azepanone-based inhibitors of human and rat cathepsin K. J. Med. Chem..

[176] Ochi Y., Yamada H., Mori H., Nakanishi Y., Nishikawa S., Kayasuga R., Kawada N., Kunishige A., Hashimoto Y., Tanaka M. (2011). Effects of ONO-5334, a novel orally-active inhibitor of cathepsin K, on bone metabolism. Bone.

[177] Riva L., Yuan S., Yin X., Martin-Sancho L., Matsunaga N., Pache L., Burgstaller-Muehlbacher S., De Jesus P.D., Teriete P., Hull M.V. (2020). Discovery of SARS-CoV-2 antiviral drugs through large-scale compound repurposing. Nature.

[178] Altmann E., Renaud J., Green J., Farley D., Cutting B., Jahnke W. (2002). Arylaminoethyl amides as novel non-covalent cathepsin K inhibitors. J. Med. Chem..

[179] Wilson S.R., Peters C., Saftig P., Brömme D. (2009). Cathepsin K activity-dependent regulation of osteoclast actin ring formation and bone resorption. J. Biol. Chem..

[180] Palmer J.T., Bryant C., Wang D.X., Davis D.E., Setti E.L., Rydzewski R.M., Venkatraman S., Tian Z.Q., Burrill L.C., Mendonca R.V. (2005). Design and synthesis of tri-ring P3 benzamide-containing aminonitriles as potent, selective, orally effective inhibitors of cathepsin K. J. Med. Chem..

[181] Catalano J.G., Deaton D.N., Long S.T., McFadyen R.B., Miller L.R., Payne J.A., Wells-Knecht K.J., Wright L.L. (2004). Design of small molecule ketoamide-based inhibitors of cathepsin K. Bioorg. Med. Chem. Lett..

[182] Thompson S.K., Halbert S.M., Bossard M.J., Tomaszek T.A., Levy M.A., Zhao B., Smith W.W., Abdel-Meguid S.S., Janson C.A., D’Alessio K.J. (1997). Design of potent and selective human cathepsin K inhibitors that span the active site. Proc. Natl. Acad. Sci. USA.

[183] Crane S.N., Black W.C., Palmer J.T., Davis D.E., Setti E., Robichaud J., Paquet J., Oballa R.M., Bayly C.I., McKay D.J. (2006). Beta-substituted cyclohexanecarboxamide: A nonpeptidic framework for the design of potent inhibitors of cathepsin K. J. Med. Chem..

[184] Goričan T., Ciber L., Petek N., Svete J., Novinec M. (2021). Synthesis and kinetic characterization of hyperbolic inhibitors of human cathepsins K and S based on a succinimide scaffold. Bioorg. Chem..

[185] Zeng G.Z., Pan X.L., Tan N.H., Xiong J., Zhang Y.M. (2006). Natural biflavones as novel inhibitors of cathepsin B and K. Eur. J. Med. Chem..

[186] Qiu Z.C., Dong X.L., Dai Y., Xiao G.K., Wang X.L., Wong K.C., Wong M.S., Yao X.S. (2016). Discovery of a New Class of Cathepsin K Inhibitors in *Rhizoma Drynariae* as Potential Candidates for the Treatment of Osteoporosis. Int. J. Mol. Sci..

[187] Tavares F.X., Boncek V., Deaton D.N., Hassell A.M., Long S.T., Miller A.B., Payne A.A., Miller L.R., Shewchuk L.M., Wells-Knecht K. (2004). Design of potent, selective, and orally bioavailable inhibitors of cysteine protease cathepsin k. J. Med. Chem..

